# Forest carbon allocation modelling under climate change

**DOI:** 10.1093/treephys/tpz105

**Published:** 2019-11-21

**Authors:** Katarína Merganičová, Ján Merganič, Aleksi Lehtonen, Giorgio Vacchiano, Maša Zorana Ostrogović Sever, Andrey L D Augustynczik, Rüdiger Grote, Ina Kyselová, Annikki Mäkelä, Rasoul Yousefpour, Jan Krejza, Alessio Collalti, Christopher P O Reyer

**Affiliations:** 1 Czech University of Life Sciences, Prague, Faculty of Forestry and Wood Sciences, Kamýcká 129, 16500 Praha-Suchdol, Czech Republic; 2 Technical University Zvolen, Forestry Faculty, T. G. Masaryka 24, 96053 Zvolen, Slovakia; 3 The Finnish Forest Research Institute - Luke, PO Box 18 (Jokiniemenkuja 1), FI-01301 Vantaa, Finland; 4 Università degli Studi di Milano, DISAA. Via Celoria 2, 20132 Milano, Italy; 5 Croatian Forest Research Institute, Department for forest management and forestry economics, Cvjetno naselje 41, 10450 Jastrebarsko, Croatia; 6 University of Freiburg, Tennenbacher Str. 4 (2. OG), D-79106 Freiburg, Germany; 7 Institute of Meteorology and Climate Research (IMK-IFU), Karlsruhe Institute of Technology, Garmisch-Partenkirchen, Germany; 8 Global Change Research Institute CAS, Bělidla 986/4a, 603 00 Brno, Czech Republic; 9 University of Helsinki, Department of Forest Science, Latokartanonkaari 7, P.O. Box 27, 00014 Helsinki, Finland; 10 National Research Council of Italy, Institute for Agriculture and Forestry Systems in the Mediterranean (CNR-ISAFOM), 87036 Rende, Italy; 11 Department of Innovation in Biological, Agro-food and Forest Systems, University of Tuscia, 01100 Viterbo, Italy; 12 Potsdam Institute for Climate Impact Research, Telegraphenberg, PO Box 601203, D-14473 Potsdam, Germany

**Keywords:** carbon partitioning, fixed ratio, model calibration, mycorrhiza, natural disturbances, natural resources, nonstructural carbohydrates, repair and defence function, reproduction, temporal resolution

## Abstract

Carbon allocation plays a key role in ecosystem dynamics and plant adaptation to changing environmental conditions. Hence, proper description of this process in vegetation models is crucial for the simulations of the impact of climate change on carbon cycling in forests. Here we review how carbon allocation modelling is currently implemented in 31 contrasting models to identify the main gaps compared with our theoretical and empirical understanding of carbon allocation. A hybrid approach based on combining several principles and/or types of carbon allocation modelling prevailed in the examined models, while physiologically more sophisticated approaches were used less often than empirical ones. The analysis revealed that, although the number of carbon allocation studies over the past 10 years has substantially increased, some background processes are still insufficiently understood and some issues in models are frequently poorly represented, oversimplified or even omitted. Hence, current challenges for carbon allocation modelling in forest ecosystems are (i) to overcome remaining limits in process understanding, particularly regarding the impact of disturbances on carbon allocation, accumulation and utilization of nonstructural carbohydrates, and carbon use by symbionts, and (ii) to implement existing knowledge of carbon allocation into defence, regeneration and improved resource uptake in order to better account for changing environmental conditions.

## Introduction

Process-based models are widely and intensively used for simulating long-term tree and/or forest stand growth (Bohn et al. 2014, Lonsdale et al. 2015), as well as for forecasting carbon (C) and vegetation dynamics using different climate scenarios (Peters et al. 2013, Gutiérrez et al. 2014, Sánchez-Salguero et al. 2016, Collalti et al. 2018), because they can predict water, C and nutrient flow within ecosystems. However, our understanding of the processes governing these flows is patchy (Garcia et al. 2016), with some being understood in much more detail than others. Carbon accumulation in structural and nonstructural components of forests depends on a variety of linked processes such as photosynthesis, respiration and C allocation into different compartments, including those for defence and reproduction (Xia et al. 2017). In particular, C allocation is, due to the incomplete knowledge of the underlying mechanisms that lead plants to steer C to one pool rather than to another, often oversimplified (Franklin et al. 2012, Mäkelä 2012), and considered as a major weakness of models (Le Roux et al. 2001, Richardson et al. 2015).

Carbon allocation of forest ecosystems has a critical role in the C exchange between the atmosphere and biosphere (Litton et al. 2007), and it is regarded as one of the most important plant adaptation mechanisms to environmental changes (Yan et al. 2016). Although the processes driving C partitioning to individual tree organs are still not thoroughly understood, experimental results suggest that C allocation depends on species, environmental conditions, stand structure, phenology, ontogeny and many other factors (Litton et al. 2007, Ryan et al. 2010, Poorter et al. 2011, Franklin et al. 2012, Vicca et al. 2012, de Kauwe et al. 2014, Li et al. 2016, Collalti and Prentice 2019). The C that trees allocate to woody structural components has longer residence time compared with what is allocated to leaves and fine roots (Campioli et al. 2008). Hence, if the ratio between fast and slow turnover compartments changes in response to altered resource availability and stress intensity, future predictions of C feedbacks between biosphere and atmosphere that do not account for this change may be biased (Friend et al. 2013, Lehtonen and Heikkinen 2015). Therefore, sophisticated C allocation modelling approaches are required to better understand the effects of changes in climate, air chemistry and forest management on terrestrial ecosystems. It should be noted, however, that the degree to which allocation processes need to be accounted for depends on the scope of the model application. For some particular research questions addressing only forests under steady state, modelling allocation shifts might not be a priority.

In the presented study we analyse the results from a questionnaire-based survey of 31 models operating from forest stand-scale to global levels. Our specific objectives are (i) to identify the dominant forest C allocation modelling approaches currently used in models simulating forest dynamics and (ii) to highlight identified gaps and provide examples on how to improve C allocation modelling in the context of climate change. The information should primarily help not only modellers to identify deficits and improve C allocation modules responsive to changing environmental conditions but also researchers involved in interpreting and using model results to better understand which models are useful for a particular purpose.

## Materials and methods

In our study, we adopted a broad definition of the term C allocation presented by Litton et al. (2007) encompassing both the pattern of biomass distribution among individual tree components and the process of C partitioning, i.e., the flux of C to a particular tree component per unit time defined as biomass or pool increment.

### Questionnaire survey and database creation

The questionnaire (see Supplementary A available as Supplementary Data at *Tree Physiology* Online) was prepared by the working group ‘Carbon allocation’ within the European Cooperation in Science and Technology (COST) Action network project ‘Towards robust PROjections of European FOrests UNDer climate change’ (PROFOUND FP1304) as a web-based survey. It consisted of both open-ended and closed-ended questions (Q) divided into three main parts focusing on the general description of the whole modelling system (14 questions), C allocation model implemented in the modelling system (25 questions) and reference sources (11 questions). The principles and the types of C allocation models were taken from the previous works dealing with C allocation modelling in forests (Lacointe 2000, Fabrika and Pretzsch 2011, Franklin et al. 2012, de Kauwe et al. 2014).

The survey was distributed by email to the participants of PROFOUND as well as a related COST Action networking project called ‘Climate Change Manipulation Experiments in Terrestrial Ecosystems—Networking and Outreach’ (ClimMani), the INTERFACE research coordination network, and further forwarded to relevant model developers and model users based on personal contacts of participants. In total, we invited approximately 260 scientists worldwide. Participation in the survey was voluntary. The survey was open from 11 November 2016, to 31 January 2017. Since non-European researchers were not present during the meetings of the COST Actions, during which the questionnaire was developed and presented, the response rate from those regions was lower.

In total, we gathered 40 responses with information about C allocation modelling approaches implemented in 31 different models (Table 1) from 16 countries (see Figure S1 available as Supplementary Data at *Tree Physiology* Online). This number of models reflects the number of complex vegetation based models found in preceding studies focusing on a similar pool of models (Fontes et al. 2010). The applied modelling approaches varied from the viewpoint of temporal, spatial and modelled units as defined by Fabrika and Pretzsch (2011) (see Figure S2 available as Supplementary Data at *Tree Physiology* Online).

**Table 1:** List of examined vegetation models in this study. Modelling approach refers to a broad specification of how processes are modelled by the whole modelling system; in the case of a hybrid approach, several modelling concepts are combined, while the dominant modelling concept is presented in table. Carbon allocation types are defined in Table 2.

Name of the model	Whole modelling system		Applied types of carbon allocation	References
	Modelling approach	Dominant modelling concept		
3D-CMCC FEM	Hybrid	Process-based	Allometry and resource limitation	Lüdeke et al. (1994), Arora and Boer (2005), Collalti et al. (2014, 2016, 2018, 2019*a*) and Marconi et al. (2017)
3PG-BW	Hybrid	Process-based	Allometry and resource limitation	Landsberg and Waring (1997)
ANAFORE	Hybrid	Process-based	Pipe model, resource limitation and source–sink model	Deckmyn et al. (2008)
BALANCE	Hybrid	Process-based	Pipe model, source–sink model and root–shoot functional balance	Rötzer et al. (2010, 2012), Grote and Pretzsch 2002
BASFOR	Hybrid	Process-based	Fixed ratios, resource limitation, source–sink model and root–shoot functional balance	Van Oijen et al. (2005)
Biome-BGC	Process-based	Process-based	Fixed ratios	Thornton et al. (2005)
Biome-BGCMuSo	Process-based	Process-based	Fixed ratios	Running and Hunt (1993) and Hidy et al. (2016)
CARAIB	Process-based	Process-based	Fixed ratios	Warnant et al. (1994)
CASTANEA	Process-based	Process-based	Allometry, pipe model and resource limitation	Dufrêne et al. (2005) and Guillemot et al. (2016)
CENTURY	Process-based	Process-based	Fixed ratios and resource limitation	Parton et al. (1987) and Allister et al. (1993)
Community Land Model (CLM4.5)	Hybrid	Process-based	Allometry and resource limitation	Oleson et al. (2013) and Fan et al. (2015)
CoupModel	Hybrid	Process-based	Allometry, fixed ratios, optimal response, resource limitation and transport resistance	Eckersten and Jansson (1991), de Willigen (1991), Jansson and Karlberg (2004) and Svensson et al. (2008)
ED2	Hybrid	Process-based	Allometry, fixed ratios and pipe model	Medvigy et al. (2009) and Hurtt et al. (2013)
FORESEE (4C)	Hybrid	Process-based	Allometry and pipe model	Bugmann et al. (1997) and Lasch-Born et al. (2019)
ForGEM	Empirical	Empirical	Allometry	Kramer et al. (2008), Kramer and van der Werf (2010) and Kramer et al. (2015),
FORMIND	Process-based	Process-based	Allometry	Bohn et al. (2014)
GO+	Hybrid	Process-based	Allometry, optimal response and resource limitation	Loustau (2010)
GO+TreeStabd	Hybrid	Structural	Allometry	Loustau et al. (2005)
GOTILWA+	Process-based	Process-based	Pipe model and source–sink model	Shinozaki et al. (1964) and Keenan et al. (2009)
Heterofor	Hybrid	Empirical	Allometry and root–shoot functional balance	Jonard and André (2018)
iLand	Hybrid	Process-based	Allometry and root–shoot functional balance	Seidl et al. (2012)
Klein & Hoch	Process-based	Process-based	Source–sink model	Klein and Hoch (2014)
LANDIS-II	Hybrid	Process-based	Allometry, fixed ratios and resource limitation	Scheller et al. (2011)
LandscapeDNDC	Hybrid	Process-based	Pipe model and source–sink model	Grote (1998), Grote and Reiter (2004) and Grote et al. (2011)
LIGNUM	Hybrid	Process-based	Allometry, pipe model and source–sink model	Sievänen et al. (2008) and Perttunen et al. (1998)
LPJ-GUESS	Hybrid	Process-based	Allometry, fixed ratios, pipe model, resource limitation and root–shoot functional balance	Smith et al. (2001), Sitch et al. (2003) and Smith et al. (2014)
ORCHIDEE-CAN	Hybrid	Process-based	Allometry, pipe model and source–sink model	Naudts et al. (2015)
PICUS	Hybrid	Process-based	Allometry, pipe model and source–sink model	Lexer and Hönninger (2001), Seidl et al. (2005), Seidl et al. (2007) and Seidl et al. (2009)
PnET	Hybrid	Empirical	Fixed ratios and pipe model	Aber and Federer (1992)
SIBYLA	Empirical	Empirical	Allometry	Fabrika (2005), Fabrika and Ďurský (2006)Fabrika and Pretzsch (2011)
TreeMig	Hybrid	Process-based	Fixed ratios	Bugmann (1994) and Lischke et al. (2006)

The collected responses were checked for consistency and stored in a Microsoft Access database. In the case of ambiguous replies, these were cross-checked with references and model developers and/or users who had filled in the questionnaire.

### Model complexity ranking

To perform a quantitative model intercomparison, we analysed the complexity of C allocation models based on individual questions presented in the second part of the questionnaire (see Supplementary A available as Supplementary Data at *Tree Physiology* Online). Under the term ‘complexity’, we understand the level of detail applied within a model to describe the behaviour of the system including its inter-dependencies. Complexity was quantified in four different ways depending on the underlying question: (i) the principles and types of allocation modelling (Q 2.1 and 2.2, see Supplementary A available as Supplementary Data at *Tree Physiology* Online) and their temporal and spatial scales (Q 2.3 and 2.4) were rated starting from 1, which indicated the simplest approaches and the largest scales of time and space, to the question-specific maximum (5 for principles and a spatial scale, 7 for a temporal scale and 10 for types of C allocation modelling), which represented the most complex approaches and the finest temporal and spatial scales; (ii) each individual answer on the presence of variables affecting C allocation (Q 2.5), compartments (Q 2.6), priority of C allocation (Q 2.8.1–2.8.5), model sensitivity (Q 2.9; see Supplementary A available as Supplementary Data at *Tree Physiology* Online) was rated with a value of 1; (iii) each answer on the presence of constant parameters (Q 2.7) was rated with a value of −1; and (iv) yes/no answers (Q 2.8, 2.10, 2.13) were rated with 1 or 0, respectively. In the case of multiple questions (e.g., Q 2.5 or Q 2.7), the score for the question was calculated by summing up the values for all the entries of the particular question. Afterwards, to ensure the same scale of the complexity measure for all questions the total score of each question was rescaled in the range 0–1, with 1 representing the maximum attainable score. Hence, values close to 0 suggest low complexity of C allocation modelling and values close to 1 indicate high complexity. This is in line with Jin et al. (2016), who stated that complex models closely couple environmental conditions and physiological processes, involve more variables than simpler models and operate at finer temporal scales. The obtained complexity values were then further used in the analysis of gaps in C allocation modelling.

### Analysis of the gaps in carbon allocation modelling

Most frequent gaps in the representation of C allocation in forest growth models identified by the respondents (Q 2.13.1 in Supplementary A) were analysed in three steps:

- (i) Identification of the gap
- (ii) Evidence to prove the gap
- (iii) Approaches and examples to overcome the gap

The existence of the gap was further examined using the responses on related questions from the second part of the questionnaire (Q 2.1 to 2.12). We were primarily concerned with the frequency of the gap, i.e., in how many models the identified problem may potentially occur. The evidence of the identified gaps was justified by a literature review to independently confirm the relevance of each gap for accurate modelling of C allocation using published empirical evidence. Finally, we examined possible modelling approaches to overcome the identified gaps, either from the models specified in the questionnaire or from other existing modelling approaches in the literature. For the literature review, we used the databases of Elsevier Scopus©, ISI Web of Knowledge©, CAB Abstract© and Google Scholar©. The material was selected by searching for the term ‘carbon allocation’ and its synonyms identified by Litton et al. (2007) in combination with the terms ‘model’ or ‘modelling’ in the title, abstract and/or keywords of published papers in English.

## Results

**Figure 1.**
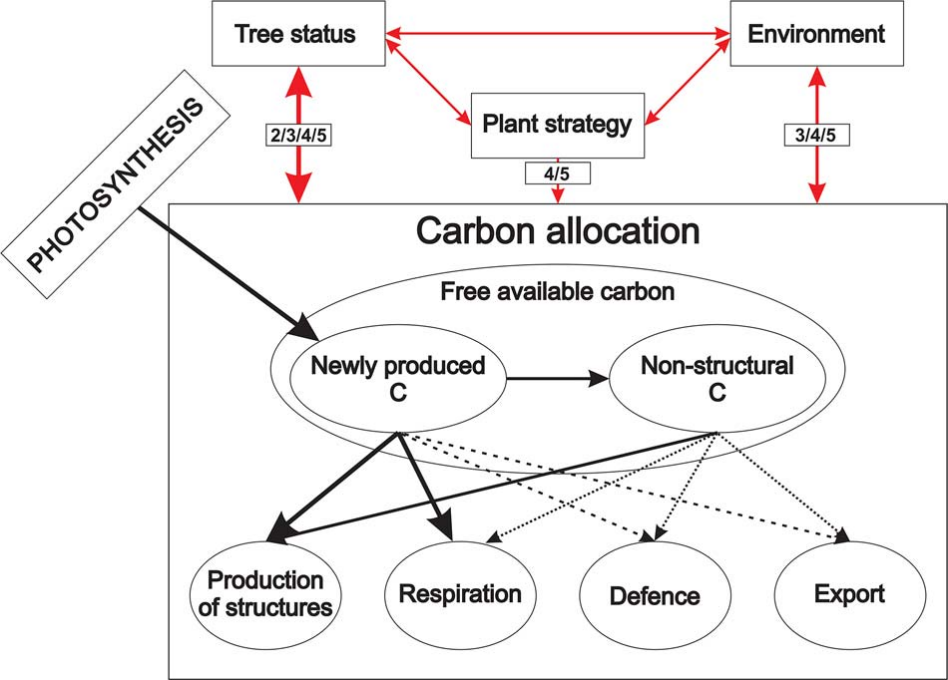
Scheme of carbon allocation in plants. Black arrows inside the box represent carbon pathways; red arrows outside the box show the directions of impacts. Thick arrows indicate that all examined models simulate the particular pathway; moderately thick arrows show that only a part of models account for the movement, and dashed arrows represent the links that were experimentally proven, but were not explicitly simulated by any of the models included in the analysis. The numbers in small boxes indicate which carbon allocation principle is able to account for this influence (2, functional relationship; 3, functional balance; 4, eco-evolutionarily-based; 5, thermodynamic principle).

As Franklin et al. (2012) pointed out, C allocation is not a process but an outcome of several different processes. Photosynthates produced by plants are allocated to physiologically different parts of plant functioning (Figure 1). The C assigned to plant structures is used for the production of new structural tissues of both vegetative and generative plant organs to ensure resource uptake (leaves and fine roots), plant functionality and support (stem, branches and coarse roots), and reproduction (flowers, fruits and seeds). In order to keep the plant functioning, some portion of available C is respired. For the protection of already captured resources, some carbohydrates are used as mechanical or chemical defence. Plants also export some portion of fixed C into mycorrhiza or into the soil in the form of root exudates to increase their nutrient uptake. A portion of photosynthates is stored as nonstructural compounds, mainly starch and sugars, which represent plant reserves that can be used in future for any of the above-mentioned reasons. Plant allocation strategy determines which C allocation pool is favoured at a particular point in time. The choice of the strategy and subsequently the proportions of C allocated to individual parts are influenced by the actual state of the plant (age, size, etc.), by the surrounding environment (water, nutrients, temperature, etc.) and by disturbances including management. From the point of plant survival, all pathways are indispensable. However, in the models they are unequally represented (Figure 1). In the context of climate change, under which disturbances and/or adverse environmental conditions have become more frequent (Seidl et al. 2017), causing shifts in allocation patterns (Litton and Giardina 2008), accounting for underrepresented C pathways in models may be crucial.

### Approaches to carbon allocation modelling

**Figure 2.**
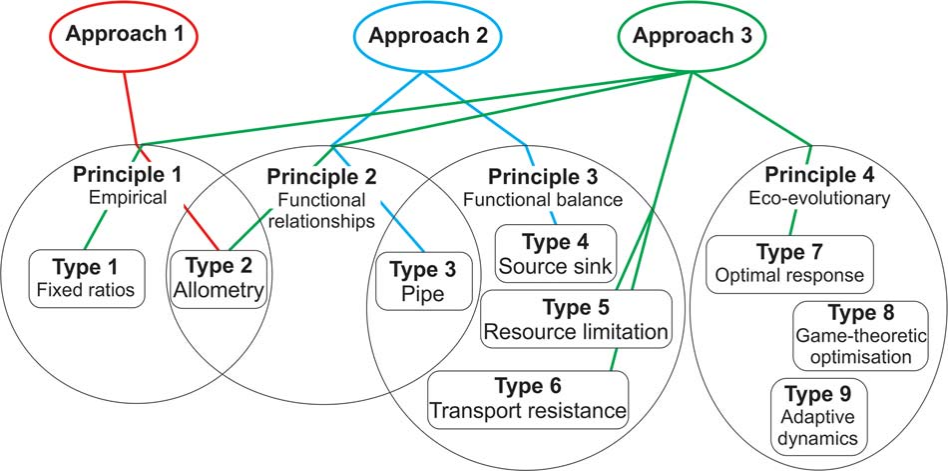
Examples of approaches applied in vegetation models using different principles and types of carbon allocation modelling: Approach 1 applied in SIBYLA, Approach 2 in LANDSCAPE DNDC and Approach 3 in CoupModel. Approaches 2 and 3 are examples of combinations of several carbon allocation types.

**Figure 3.**
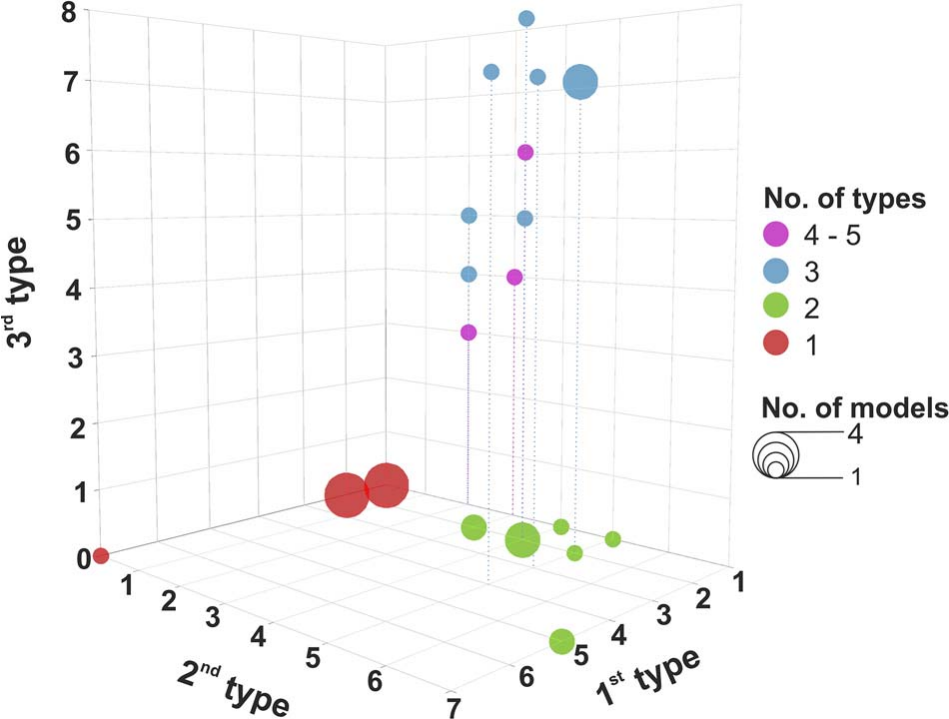
Combinations of different types of carbon allocation modelling in the investigated vegetation models. Numbers on axes represent individual types of carbon allocation modelling as follows: 1, fixed ratios; 2, allometry; 3, root–shoot functional balance; 4, resource limitation; 5, pipe model; 6, transport resistance; 7, source–sink model; 8, optimal response. The size of the bubble indicates the number of models from our database that use a particular type or a combination of types for modelling carbon allocation, with the smallest size representing one model and the biggest size representing four models. Red colour indicates that only one type of carbon allocation modelling has been applied, green colour indicates the combination of two types, blue colour stands for the combination of three types and purple colour for four or five types of carbon allocation modelling, while only the first three types are explicitly presented on the axes.

Investigated models differed in applied C allocation modelling approaches. Fifteen models used a single principle of C allocation modelling as defined by Franklin et al. (2012) (Table 2), while 16 were based on a combination of at least two principles. Out of these, 11 models combined two principles, 4 models combined three principles and CoupModel combined four different principles of C allocation modelling (Figure 2). The frequency of applying individual principles and/or types decreased with their complexity (Table 2; Figure 3). Empirically defined C allocation was most commonly used (61% of models), followed by the principles of functional relationship and functional balance (Table 2). Eco-evolutionary-based types of C allocation modelling were used in three models (CLM 4.5, CoupModel and GO+), while the thermodynamic principle was not used in any (Table 2).

**Table 2:** Description of principles and types of carbon allocation modelling and the frequency of their usage in examined vegetation models.

ID of carbon allocation principle	Principle of carbon allocation modelling	Basic description	Computation efficiency	Variation of carbon allocation with size/age	Variation of carbon allocation with environment	Feedback between plant’s strategy and environment	Number of models
1	Empirical	Carbon allocation is based on constant statistical relationships among individual organs.	High	No	No	No	19
2	Functional relationship	Carbon allocation is defined by allometric functions describing relationships among plant organs.	High	Yes	No	No	16
3	Functional balance	Carbon is allocated to maintain internal balance between organs according to an optimum internal status of resource or element ratio.	Moderate	Yes	Yes	No	16
4	Eco-evolutionarily-based	Carbon is allocated in order to maximize a fitness proxy.	Low	Yes	Yes	Yes	3
5	Thermodynamic	Carbon is allocated in order to maximize entropy or entropy production.	Moderate	Yes	Yes	Yes	0
	Type of carbon allocation modelling						
1	Fixed ratios	Fixed fractions of assimilated carbon are allocated to individual organs.	High	No	No	No	10
1 (2)	Allometry	Carbon is allocated to a particular organ according to mass and size relationships.	High	Yes	No	No	19
2 (3)	Pipe model	Carbon is allocated in order to provide the (sapwood) conductance necessary to support foliage.	High	Yes	No/yes	No	12
3	Root–shoot functional balance	Carbon is allocated to individual organs to ensure a balanced supply of resources from foliage and fine roots.	Moderate	Yes	Yes	No	6
3	Resource limitation	Allocation of assimilated carbon to individual organs is driven by the most limiting source to growth.	Moderate	No/yes	Yes	No	12
3	Source–sink model	Allocation of assimilated carbon to individual organs is driven by the demands of individual organs and the availability of assimilates.	Moderate	Yes	Yes	No	9
3	Transport resistance	Allocation of assimilated carbon is controlled by concentration gradients of elements/compounds between plant parts.	Low	Yes	Yes	No	1
4	Optimal response	Selects an optimal allocation strategy that maximizes a predefined goal (fitness proxy) when there is a significant competition only for one resource.	Low	Yes	Yes	No	2
4	Game-theoretic optimization	Selects an optimal allocation strategy that maximizes a predefined goal (fitness proxy) when there is a significant competition for more than one resource.	Low	Yes	Yes	Yes	0
4	Adaptive dynamics	Selects an optimal allocation strategy that maximizes a goal (fitness proxy), which is dynamically selected.	Low	Yes	Yes	Yes	0
5	Maximum entropy production	Selects the most probable allocation strategy that maximizes entropy under given environmental and internal constraints.	Moderate	Yes	Yes	Yes	0
5	Maximum entropy	Predicts the most probable allocation strategy and the frequency distribution of different strategies (allocation patterns) around the most probable strategy under given environmental and internal constraints.	Moderate	Yes	Yes	Yes	0

### Identified gaps in carbon allocation modelling

Model developers and users identified 24 specific problems related to C allocation modelling. The most commonly identified problems were (i) usage of fixed ratios despite known natural dynamics of C allocation, lack of direct sensitivity of C allocation modelling (ii) to environmental conditions and (iii) to natural disturbances, (iv) missing pools that may trigger C losses under environmental changes or function as a buffer to withstand stress conditions, (v) allocation time steps that are too large to model the dynamics of resource acquisition and (vi) lack of data for calibration and validation of C allocation procedures. These issues are of particular importance in the context of ongoing climate change, which may cause unprecedented shifts in environmental conditions that drive ecosystem and plant processes including C allocation (DeLucia et al. 2000). Below we specifically analyse each gap using the two first steps defined in the section Analysis of the gaps in carbon allocation modelling. The approaches to overcome the gaps are summarized at the end of the results.

**Figure 4.**
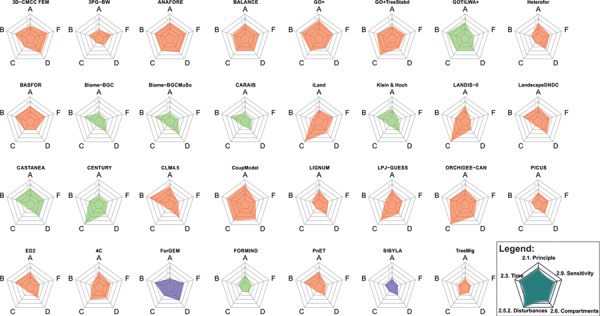
Relative complexity of the models reviewed in this study. Values close to 1 indicate high complexity of the model, while values close to 0 indicate low complexity. The five dimensions of the spider plot refer to individual questions on carbon allocation modelling posed in the questionnaire (A, Q 2.1 principle of carbon allocation modelling; B, Q 2.3 time step of the carbon allocation model; C, Q 2.5.2 disturbances that affect carbon allocation; D, Q 2.6 individual compartments for carbon allocation; F, Q 2.9 sensitivity of carbon allocation algorithm to individual factors). The colours indicate the modelling approach of the whole modelling system (orange, hybrid; green, process-based; purple, empirical).

To analyse model complexity from the viewpoint of the gaps identified by model respondents, we visualized the values of relative complexity for each model that were derived from the responses to those questions related to the analysed gaps (five questions) following the methodology in the section Model complexity ranking. The results indicate that few models are complex in all five characteristics tested here, i.e., some models use more complex principles of C allocation modelling, while other models operate at a finer temporal scale, and some others account for the impact of disturbance factors in greater detail (Figure 4).

### The use of fixed ratios for carbon allocation modelling

#### Identification of the gap

Modelling C allocation using ‘fixed ratios’ assumes that compartment fractions, C allocation ratios and/or growth proportions are held constant (Franklin et al. 2012). These parameters may be set depending on specific environmental conditions, e.g., vegetation group/biome/plant functional types/tree species, soil water and nutrient status, etc., but they do not change in response to phenology, stand development or varying environmental conditions and natural disturbances.

More than a half of the investigated models (18 models, 58%) applied fixed C allocation to a certain extent (Q 2.2 and Q 2.7, see Supplementary A available as Supplementary Data at *Tree Physiology* Online). Carbon allocation based solely on fixed ratios was used in four models, while others used a hybrid modelling approach that combined fixed allocation with one or more other modelling types, usually allometry, resource limitation or pipe model (Figures 2 and 3). Models with fixed ratios represent an oversimplification of the underlying mechanisms (Figure 1; Collalti et al. 2019*a*). Since climate change is expected to induce changes in forests, using fixed coefficients is evidently a shortcoming when modelling forest development (Litton et al. 2007, Ostrogović Sever et al. 2017, Collalti et al. 2019*b*) even with the models combining fixed ratios with more sophisticated approaches (de Kauwe et al. 2014).

#### Evidence to prove the gap

Although fixed C allocation ratios could be applicable in special cases, such as large-scale modelling of forests in a steady state (see CLM 4.5), for most purposes C allocation appears dynamic, involving different plant processes driven by a variety of environmental factors (Wardlaw 1990). Its dynamics can be synthesized into: (i) seasonal—due to phenology (White et al. 1997, Caldararu et al. 2014, Collalti et al., 2014, Delpierre et al. 2015, Schiestl-Aalto et al. 2015, Collalti et al., 2016, Marconi et al. 2017); (ii) periodical—during stand development due to age- or size-related parameters or processes (Franklin et al. 2012), e.g., age-dependent root-to-shoot ratio (Genet et al. 2009), age-dependent partitioning of C into foliage and wood (Litton et al. 2007, Valentine and Mäkelä 2012), tree height-related dynamic of nonstructural carbohydrates (NSC) (Sala and Hoch 2009), masting dynamics (Vacchiano et al. 2018; see Chapter Missing pools and repair functions), stand density (Poorter et al. 2011, Krejza et al. 2013), competition (Vanninen and Mäkelä 2005); and (iii) long term—due to direct sensitivity of C allocation processes to environmental conditions (Poorter et al. 2011, Chapter Direct sensitivity of carbon allocation to environmental conditions) and natural disturbances (Running 2008, Chapter Missing pools and repair functions).

The most pronounced effect of climate change on C allocation is expected to be evident in its long-term dynamics due to direct sensitivity of C allocation to environmental conditions. Nevertheless, climate change can also indirectly alter seasonal C allocation dynamics through shifts in plant phenology (Cleland et al. 2007). Moreover, under climate change, increasing plant respiration may push plants to allocate more C to reserves than to structural growth (Collalti et al. 2018), affecting also periodical NSC dynamics.

The problem of ‘fixed ratios’ is also evident through fixed growth proportions, i.e., growth derived from C assimilation, an approach that is commonly used in process-based models (White et al. 1997, Mäkelä et al. 2000, Caldararu et al. 2014). Nevertheless, it is known that growth may be uncoupled from net photosynthesis (Fatichi et al. 2014, Körner 2015), relying more on C storage and being more sensitive to temperature, nutrient and water limitation than photosynthesis (Muller et al. 2011, Schiestl-Aalto and Mäkelä 2017).

### Direct sensitivity of carbon allocation to environmental conditions

#### Identification of the gap

**Figure 5.**
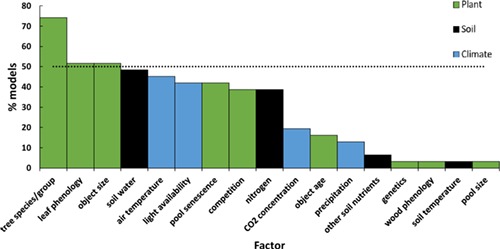
Percentage of models that account for the impact of different factors on carbon allocation (dashed line represents 50% of models).

Including direct environmental controls of C allocation in models is fundamental if the aim is to simulate ecosystem dynamics under the ongoing climate change. We identified 17 factors that influence simulated C allocation in the examined models, out of which 8 represented environment, i.e., climate and soil (Q 2.9, see Supplementary A available as Supplementary Data at *Tree Physiology* Online) (Figure 5). The factors affect the dynamics of tree growth, the contribution of each tree component to autotrophic respiration and the C transfer to the rhizosphere. In particular, the latter point has been highlighted since it is driven by changes in the root–shoot ratio (e.g., Litton and Giardina 2008) and in lifespan and decomposition rates of tree components (Körner 2003, Epron et al. 2012b).

The analysis revealed that in 11 models no climatic or soil conditions directly affected simulated C allocation (see Figure S3B available as Supplementary Data at *Tree Physiology* Online). From the models that accounted for at least one of identified environmental conditions, most (14 models) considered air temperature, while precipitation affected C allocation only in 4 models (Figure 5). Only ANAFORE included the impact of three identified soil characteristics (soil water, nitrogen and other nutrients). Although nitrogen was the most frequently included nutrient in models, still 12 models do not simulate nitrogen cycling in ecosystems (Figure 5).

#### Evidence to prove the gap

Increasing temperature has the potential to increase C accumulation in aboveground biomass, meaning stimulation of the height growth more than the growth of stem diameter (Way and Oren 2010), while temperatures below 18 °C significantly increased the fraction of roots at the expense of stems and leaves (Usami et al. 2001, Overdieck et al. 2007, Kasurinen et al. 2012). Faster decomposition at higher temperatures releases more nutrients from the soil organic nitrogen pool, which could result in an increase of gross primary productivity caused by higher needle biomass production (Pumpanen et al. 2012). Increased nutrient availability leads to increased partitioning to aboveground parts of the tree and decreased partitioning to belowground tree parts (Litton et al. 2007, Repola 2008, Poorter et al. 2011), whereas reduced nutrient availability or drought generally favour C allocation to the root system, especially in the humid soil horizons (Friedlingstein et al. 1999, Konôpka and Lukac 2012, Hommel et al. 2016). Waterlogging also affects biomass fractions of leaves and roots, though in the opposite direction to water shortage, e.g., by favouring leaves (Poorter et al. 2011). Tree seedlings limited by magnesium reduced C allocation to roots, while phosphorus limitation favoured C allocation to roots (Ericsson 1995) or mycorrhizal symbionts (Ekblad et al. 1995). Potassium fertilization had a significant effect on C allocation favouring aboveground tree parts (Epron et al. 2011), and adding calcium resulted in higher C allocation to radial growth and reproductive processes (Halman et al. 2013). The elements of phosphorus, potassium and magnesium were found to be limiting for the production of late-successional ecosystems (Körner 2015).

Water and nutrient demands are closely connected with elevated atmospheric CO_2_, because increased photosynthetic rates in response to elevated atmospheric CO_2_ do not always enhance stem growth (Fatichi et al. 2014) but rather increase fruit production, C release into the soil (de Kauwe et al. 2014) or the amount of C allocated to NSC (Collalti et al. 2018). An increase in biomass accumulation as a result of higher atmospheric CO_2_ was observed only when sufficient nutrients were supplied (Murray et al. 2000, Franklin et al. 2012). The process of downward regulation may be accompanied by higher C sequestration into structural and conducting tissues as well as by reduction of photosynthetically active tissues (Murray et al. 2000, Rolo et al. 2015). The study on European beech and Norway spruce showed lower values of specific leaf areas when growing under enhanced levels of atmospheric CO_2_ (Rolo et al. 2015).

### Impact of disturbances on carbon allocation

#### Identification of the gap

**Figure 6.**
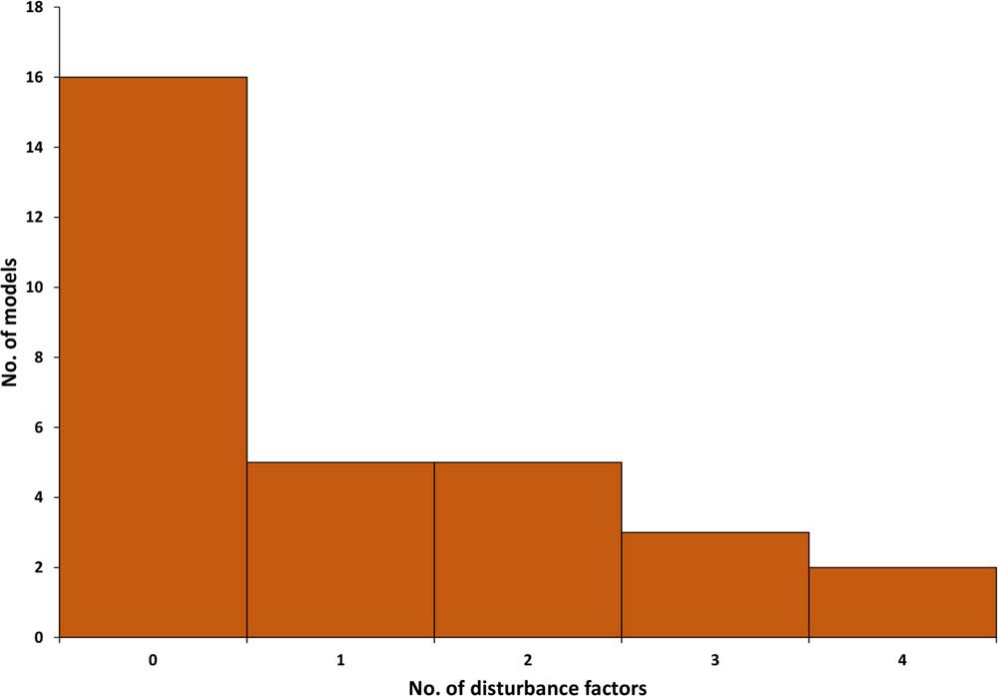
Number of natural disturbance factors (drought, fire, insects, wind or generic disturbance) affecting carbon allocation in examined models.

Climate change is a prominent reason for the observed and projected increasing frequency and intensity of disturbances (Seidl et al. 2014), which have significant impacts on forest C cycling (Hicke et al. 2012, Running 2008). Hence, modelling disturbances and the response of forest ecosystems is becoming crucial for future projections of forest dynamics. In spite of that, out of 31 models in our database, only 15 included the influence of one or several disturbances on C allocation (excluding management as a disturbance). Most of the models (10 out of 15) included one or two disturbances. The models with the highest complexity values from the viewpoint of disturbances (iLand, LANDIS-II, CENTURY and ORCHIDEE-CAN; Figure 4, C) included four different disturbance types (Figure 6).

The most commonly included disturbance effect was drought, covered by 13 models, followed by fire (6 models), wind (6 models) and insects (5 models). Two models also included ‘generic’ disturbance not associated with any specific disturbance agent (LPJ-GUESS and TreeMig). While this possibly reflects the dominance of individual disturbance agents in the different regions and forest types the models have been designed for (c.f. Reyer et al. 2017), there is increasing evidence that the interactions of disturbances are actually crucial to assess disturbance impacts under climate change (Seidl et al. 2017). No model covered the effects of other regionally important disturbances such as ice storms and pathogens.

It should be noted that many models explored here consider the effect of disturbances only indirectly, i.e., as responses of C allocation to disturbance-induced changes in light, nutrient and water availability. However, there is evidence of additional effects of drought, insect and wind damage on allocation, which are not covered by models yet. This includes a reduced hydraulic conductivity that may persist throughout years or a change in root to shoot ratios (e.g., Bansal et al. 2013). In general, even though the number of forest models that include disturbances are increasing, the disturbances are often represented by statistical approaches (Seidl et al. 2011), which complicates their integration into complex process-based models that deal with allocation mechanistically.

#### Evidence to prove the gap

Drought, insect and wind damage have direct effects on C allocation in trees. Although the reactions may be species specific, a recent meta-analysis by Eziz et al. (2017) revealed that under drought conditions the fraction of plant root mass and reserves generally increased, while the fraction of stem, leaf and reproductive biomass decreased. The process is enhanced by increasing fine root mortality under dry conditions although, at a certain threshold, fine root production decreases again (Meier and Leuschner 2008, Nikolova et al. 2010). According to Galvez et al. (2011), severe drought stress promotes the accumulation of carbohydrate reserves in roots at the expense of growth. Similarly, Liu et al. (2017) indicated an accumulation of NSC in leaves and reduced shoot and stem growth under severe summer drought conditions. However, as Hartmann and Trumbore (2016) pointed out, the accumulation of NSC occurs only in the case of short-term drought events. After the drought, plants favour root growth as a recovery strategy in order to restore root functions (Hagedorn et al. 2016). Seidl and Blennow (2012) hypothesized that post-storm stem growth reductions of the remaining trees in Sweden might be caused by allocation changes to repair root damages and produce insect defence compounds. The former mechanism has been found both in tree-pulling experiments (Nielsen and Knudsen 2004) and field data analysis (Vargas et al. 2009). Also, analyses on seedlings have shown that mechanical stimuli mimicking natural wind sways increase biomass allocation to roots (Coutand et al. 2008). Investment in insect defense compounds has been shown for mildly drought-affected trees (McDowell 2011). Defoliation is also known to cause shifts in C allocation towards new leaf production (Mayfield et al. 2005, Eyles et al. 2009, Pinkard et al. 2011, Jacquet et al. 2012) and accumulation of reserves at the expense of stem growth (Wiley et al. 2013, Piper et al. 2015). Saffell et al. (2014) showed that trees suffering from a chronic fungal disease of leaves changed their C allocation in favour of NSCs in crowns to maintain foliage growth and shoot extension in the spring. Browsing was also found to have an effect on C allocation in trees, particularly in the short term (Palacio et al. 2008,  2011, Endrulat et al. 2016).

### Missing pools and repair functions

#### Identification of the gap

**Figure 7.**
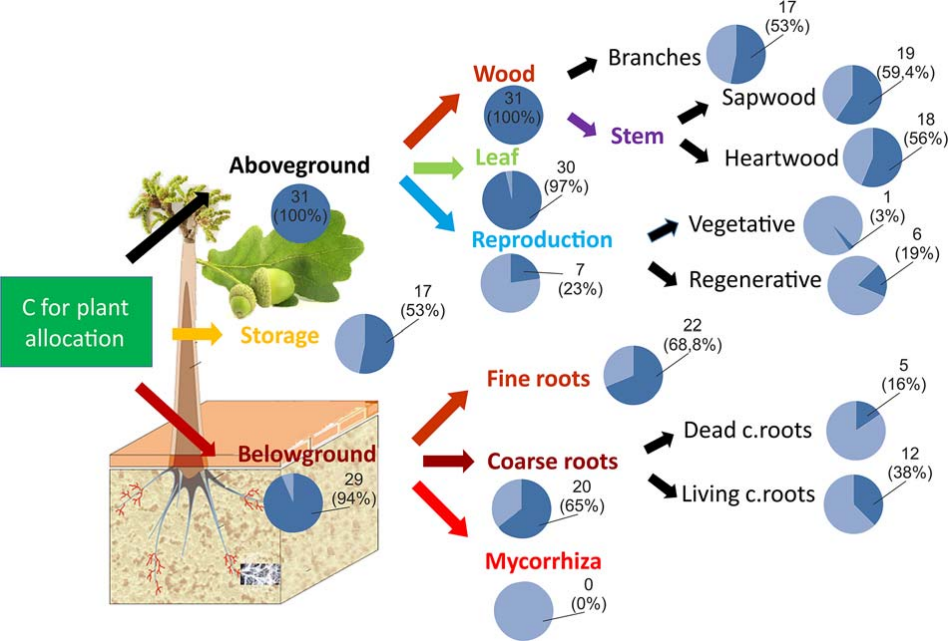
Frequency of tree compartments used in models.

Pathways of C within a plant are unequally considered in models (Figure 1). Under climate change, characterized by shifts in environmental conditions and more frequent extreme events, C allocation in under-represented plant parts or processes may be favoured to ensure the survival of an individual or population. Thus, models omitting these pathways may become incapable of providing the complete picture of C cycling in forests under novel conditions. On average the models allocated C to 6 (calculated mean of 5.8) different biomass compartments. Two models (TreeMig and FORMIND) distinguished only two compartments, while the most complex representation of biomass pools with a maximum of nine different compartments was implemented in CoupModel and 3D-CMCC FEM (Figure 4, D). The leaf compartment was included in all but one model, followed by fine roots used in 22 models and sapwood used in 19 models (Figure 7). Although the average number of compartments coincides with the number of main plant parts according to Cannell and Dewar (1994), reproductive and storage sinks were not frequently represented in the models (Figures 1 and 7).

A storage/reserve pool that represents nonstructural C is included in a half of the models; one model (LPJ-GUESS) includes a C pool for vegetative reproduction and six for sexual reproduction (Figure 7). Of these, two activate such a pool only for crops (CoupModel and CLM 4.5), one uses fixed allocation fractions for fruit production (Biome-BGCMuSo), while three use fixed fractions during defined periods (ANAFORE, ORCHIDEE-CAN and 3D-CMCC FEM).

Aside from missing pools, two more deficits regarding C allocation pools were identified: C available for defence and repair and C export, particularly the C that is provided to symbionts, i.e., mycorrhiza, which can account up to 30% of annual net primary production (NPP; Hobbie 2006, Courty et al. 2010). Defence and repair processes are important under stressful conditions and are particularly relevant for determining tree mortality. Allocated C to mycorrhiza might be seen as a part of the investment into resource acquisition by roots and are thus implicitly considered in root turnover and specific uptake parameters. However, this implicit consideration assumes that the relationship between plant and symbiont stays constant, which is not the case in a changing environment (Vargas 2009). Nevertheless, none of the models explicitly accounted either for C export to mycorrhiza or for defence and repair processes (Figures 1 and 7).

#### Evidence to prove the gap

Seed production can consume between 3% and 20% of annual gross primary production (GPP; Schaefer et al. 2008), depending on species and on interannual variability in reproductive output. In tree species with irregular fruiting patterns, peak seed years (‘masting’: Ascoli et al. 2017) may result in reductions of 40% in woody growth (Holmsgaard 1955, Eis et al. 1965, Selås et al. 2002, Monks and Kelly 2006, Drobyshev et al. 2010). This indicates that large resources are invested into the reproductive pool, governed by resource accumulation and depletion mechanisms and growth reproduction trade-offs (Hacket-Pain et al. 2015). Moreover, although masting can synchronize over large areas in response to weather-related drivers (Vacchiano et al. 2017), a huge variability in seed output and its response to the environment exists at the individual tree level (van der Meer et al. 2002, Vilà-Cabrera et al. 2014). In general, the results indicate that resource accumulation in cooler years triggers larger fruiting/masting events later on, with later warm temperatures inducing mast flowering (Sala et al. 2012*b*, Müller-Haubold et al. 2015, Abe et al. 2016, Monks et al. 2016, Pearse et al. 2016). Interestingly, it is nevertheless not the stored C but the newly produced C that is actually used for fruits and seeds (Hoch et al. 2003, 2013), which is corroborated by a frequent decline of wood growth in a masting year (e.g., Drobyshev et al. 2010, Martín et al. 2015). This indicates that full resource pools are a trigger for allocation changes rather than the source of masting. In addition, stress has been suggested to trigger seed production based on the theory that mortality-inducing events create favourable conditions for regeneration (Piovesan and Adams 2001, 2005), which however, has not always been supported by measurements (Müller-Haubold et al. 2015).

**Table 3:** Comparison of time step of the allocation model and the whole modelling system. Numbers indicate the number of models with the respective combination of time steps. Red colour indicates the same time step at both modelling levels; green colour indicates that the carbon allocation module operates at coarser temporal resolution than the whole modelling system, while blue colour indicates the opposite.

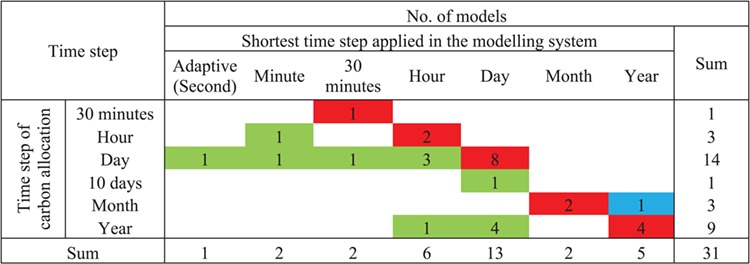

Under climate change, storage represents an important pool as it facilitates recovery processes (Hartmann 2015) after environmental disturbances (e.g., drought, fire, pathogen attacks and defoliation by insect; Barigah et al. 2013). Temperate deciduous tree species store a large amount of NSC in their stems, which could be used for stem growth for a period of 7 to 30 years (Klein et al. 2016a). For modelling purposes, NSCs are important as reserves are used not only to control their overall annual C cycle and the NPP/GPP ratio (Collalti et al. 2019*b*, Collalti and Prentice 2019), but also to repair or replace stress-related damages. This is a prerequisite to mortality estimates and also affects long-term development including delayed recovery and carry-over effects.

Similar to seed production, plants can invest up to 22% of their GPP to their fungal symbionts (Vargas 2009). The differentiation of C allocated to mycorrhiza is mainly required under changing environmental conditions (Hasselquist et al. 2016, Schiestl-Aalto et al. 2019), since climate change will significantly modify mycorrhizal diversity (Bellgard and Williams 2011), which will subsequently affect plant growth and survival. In particular, nitrogen addition, and also higher temperatures that lead to higher decomposition rates, requires differentiation between roots and fungal biomass. In contrast to the reproductive pool, which is separated from other tissues and develops under specific environmental conditions, pools for defence and repair are constitutively present and therefore need to be an integrated part of other biomass fractions (Dietze et al. 2014). Defence and repair processes are important under stressful conditions and are particularly relevant for determining tree mortality. For example, the immediate cause of death due to drought stress might be hydraulic failure (i.e., xylem cavitation) but the ability to postpone this failure may depend on the ability of the tree stabilize water conductivity, repair previous damages or build on new vessels that all depend on C supply (Sala et al. 2012a). Failure to represent this process leads to over- or underestimation of mortality, and carry-over effects of decreased growth long after the stress has ceased will be missed (Thomas et al. 2009). Similarly, air pollution leads to considerably higher damages if the constitutive defences of a leaf are exhausted (Wieser and Matyssek 2007).

### Time step of carbon allocation

#### Identification of the gap

**Figure 8.**
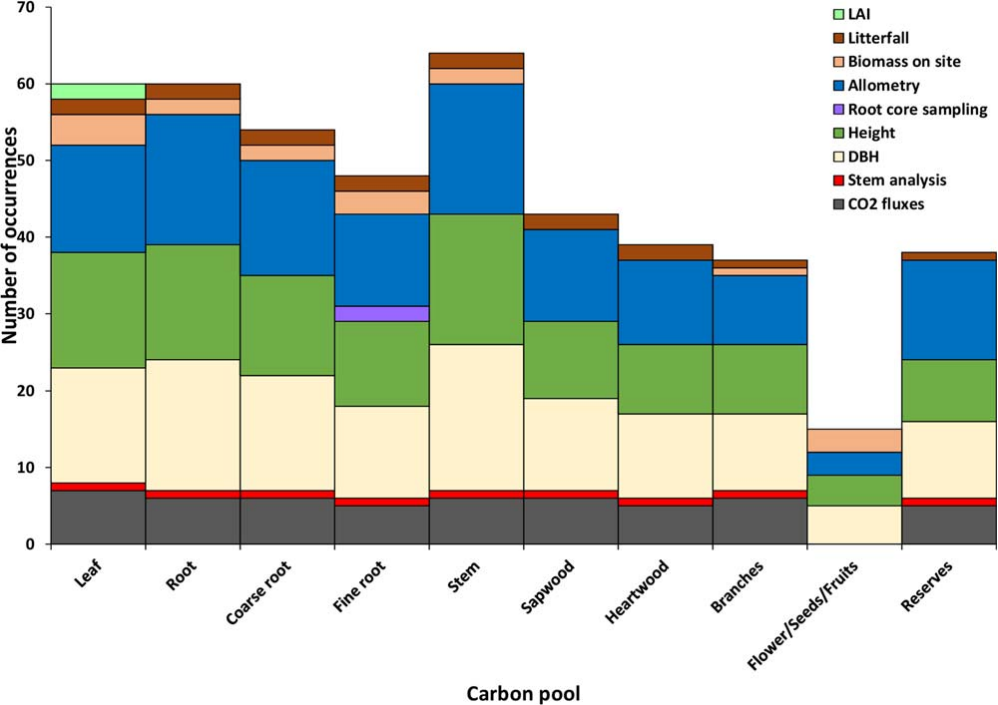
Data sources used to test the carbon allocation submodules in 24 examined models (for some models more sources of data were used). LAI, leaf area index; DBH, diameter at breast height.

The allocation of C in plants occurs at short time scales of hours and weeks (Ulrich 1993) and quickly responds to environmental changes and/or disturbances (Ferrieri et al. 2013). The results of the questionnaire revealed that C allocation models in our database worked with six different time intervals, with a year being the largest and 30 min being the smallest time step (Table 3). The daily time step was the most frequently used (45.2% models) followed by the yearly, applied in one-third of the models (Table 3). The smallest time step of 30 min was used in CLM 4.5 (Figure 4, B), as it accounts for the close linkage with highly variable atmospheric processes. Three models (CoupModel, GOTILWA+ and GO+) used a time step of 1 h. BALANCE operated at a time step of 10 days, and three models used a time step of a month (Table 3). Comparing the time step of the whole modelling system with the time step of the C allocation module, we found that 17 models used the same time steps at both modelling levels, while in 13 models the allocation module operated at a larger time step than the whole modelling system, and only in 1 model it was the other way round (Figure 8).

Models with an annual time scale (used in 29% of models) do not explicitly handle seasonal changes in C allocation due to intra-annual variations of phenology and environmental conditions, which can lead to poorly simulated fluxes also at an inter-annual scale (Vermeulen et al. 2015). In addition, most models (87%) currently do not include seasonal changes in C allocation, although the majority consider on/off of leaves for deciduous tree species. Those models that do include seasonality suffer from our general gaps of understanding of C allocation, also related to the role of C allocation to NSC.

#### Evidence to prove the gap

For more than a century, growth and biomass production have been the processes of the primary interest of foresters, while modellers have only considered growth as a result of C acquisition and allocation since the 1970s, and in particular the allocation component has not yet been thoroughly understood from physiological principles. This may be the reason why more than one-third of the models in this study use a so-called ‘top-down’ approach when simulating C allocation in ecosystems. Models operating at coarser time scales either are based on empirical relationships or use an ‘average day’ approximation (Hastings and Gross 2012). Such an approach is suitable for modelling stable systems, where slow processes at a lower temporal resolution regulate processes at higher scales (Pretzsch 2009).

Changing environmental conditions cause system instability (Scheffer et al. 2001), due to which signals from faster processes varying at higher temporal scales may become dominant and force slow processes to change (Robinson and Ek 2000, Pretzsch 2009). Models working at an annual temporal resolution often fail to capture these changes caused by novel environmental conditions (^94,131^). Finer temporal resolution enables us to examine the impact of the particular change on the analysed system (Pretzsch et al. 2015). As has already been shown above, C allocation depends on the instantaneous values of the environmental variables and their combinations (Da Silva et al. 2011). Hence, mechanistic models operating at shorter time scales are, in principle, able to provide more robust extrapolation of system behaviour under climate change (Hastings and Gross 2012). They usually include the impact of atmospheric and hydrological conditions, which are most frequently readily available at a daily resolution (Gea-Izquierdo et al. 2015). Models with seasonality often assume that the growth of a certain component is completed when its potential demand has been satisfied (Running and Gower 1991, Drouet and Pagès 2007, Gayler et al. 2007, Schippers et al. 2015), and if anything is left over, that is allocated to NSC and can be used for growth in consecutive years (i.e., ‘passive’ storage; Kozlowski 1992). However, this approach is sensitive to how the demand is determined and assumes that NSC is a passive pool, although several recent studies have demonstrated that in many cases the accumulation of NSC competes actively with growth (McDowell 2011, Sala et al. 2012; Saffell et al. 2014). Unfortunately, we still do not understand the interactions between the timing of growth, predetermined ‘growth potential’ and the environment, in order to solve these questions strictly on a physiological basis.

### Lack of data for calibration and validation of carbon allocation models

#### Identification of the gap

Arguably, the biggest challenge for modelling C allocation in forest ecosystems is data acquisition and availability. Direct measurements for the allocation of C to various tree compartments are typically resource-intensive and hard to acquire. To overcome this issue, modelling studies rely on indirect measurements of C allocation with the help of allometric relationships (e.g., Wolf et al. 2011). Despite data scarcity regarding the allocation of C in forest ecosystems, 24 out of 31 models (77%) reported in our questionnaire that their allocation modules were tested against some data. The data source used to parametrize allocation modules, however, was often not well suited to describe the underlying processes and C pools (Figure 8).

Allometric studies are dominant sources of C allocation data, especially for the stem and root pools (Figure 8). Other direct measurements of the allocation mechanism, e.g., the samples of root cores for defining fine root biomass, were reported in 2 studies out of the 24 models, indicating the need for data sources that provide a better description of below ground biomass. The accurate evaluation of the fine root compartment is critical, especially when considering the functional balance between leaves and fine roots. Moreover, only a few studies reported that the derivation of allometric relationships between tree compartments was carried out at the same sites used for calibrating and validating the C allocation models (biomass on site), whereas for the majority of studies the sources of the allometric relationships were unclear.

The use of allometric relationships based on tree height and diameter at breast height for modelling allocation into nonstructural C, reproductive structures and foliage biomass, as displayed in our results (Figure 8), may not be particularly appropriate. Traditional forest inventory collecting information on tree height and diameter is usually carried out in 1- to 5-year long intervals, and thus the data are unable to capture the short-term dynamics of the pools. For such purposes, data sources with a finer temporal scale, such as from experiments using dendrometers and microcores, are required.

#### Evidence to prove the gap

The data constraints for modelling C allocation have been widely recognized in the literature (e.g., Litton et al. 2007, Franklin et al. 2012, de Kauwe et al. 2014). While the allocation of aboveground C is fairly well understood and evaluated with allometric relationships, from which data are readily available, the dynamics of internal C allocation and the representation of belowground biomass patterns still demand investigation, as such fluxes require more detailed experiments and resource-intensive methods (Litton and Giardina 2008, Warren et al. 2011, Mildner et al. 2014). Similarly, as evidenced in our results, modelling the dynamics of NSC in reserve pools remains a major challenge. Traditionally, the evaluation of nonstructural C has been carried out through the analysis of NSC concentration in plant tissues. However, the accurate evaluation of NSC in plant tissues is a difficult task and the uncertainty related to such quantifications may be substantial (Hartmann and Trumbore 2016, Collalti et al. 2019*b*). The same caveat is highlighted by Fatichi and Leuzinger (2013), recognizing the inaccuracy of C pools and flux data as a major constraint for selecting suitable C allocation schemes and suggesting that field data collection and laboratory experiments with higher precision are key for improving C allocation modelling.

The inconsistency between datasets for evaluating C allocation patterns has also been acknowledged as an important limitation of C allocation modelling, and the harmonization of data from various sources, such as eddy covariance and forest growth data, is key for a comprehensive understanding of C allocation processes (Guillemot et al. 2015). Comparison of eddy covariance and biometric measurements data is challenging (Campioli et al. 2016, Anić et al. 2018), due to the fact that the eddy covariance method is primarily driven by canopy photosynthesis and it reflects current accumulation of atmospheric C, while biometric data represent biomass growth that uses carbohydrates from current assimilation as well as previously stored NSC (Gough et al. 2008). Linking these two datasets seems to be a promising approach for tackling the question of whole-ecosystem NSC dynamics (Gough et al. 2009). Such a link might provide valuable information on the responses of allocation patterns to environmental drivers and improve model performance.

### Approaches and examples to overcome the gaps in carbon allocation modelling

The above-discussed gaps in C allocation modelling can be solved by (i) changing and/or modifying the applied modelling approaches, (ii) integrating new components into models and (iii) direct empirical studies of C allocation. The choice of the C allocation principle/type (Table 2) predetermines the magnitude of C sequestration (e.g., Montané et al. 2017), the sensitivity to possible environmental changes (Figure 1) and model time resolution. Under climate change conditions, more complex modelling approaches would outperform simpler approaches (Table 2), since their intrinsic structure allows them to adjust in response to external impacts (Figure 1). Empirical approaches as well as a general pipe model theory assume that partitioning is in a steady state, thus they usually lack responses to environmental changes (Bugmann 1994, Franklin et al. 2012) and can be used only for a limited range of conditions (Lacointe 2000). However, in some applications of the pipe model theory, C allocation is responsive to environmental conditions, albeit just those caused by competition/stand density (Valentine and Mäkelä 2005, Mäkelä et al. 2016). Source–sink approaches (e.g., BALANCE, BASFOR and LanscapeDNDC) calculate C allocation from the actual biomass of a specific compartment. Since the compartment size is influenced by senescence (included in e.g., CASTANEA, 3D-CMCC FEM and GOTILWA), all environmental conditions that influence this process also affect allocation.

In the models that rely on functional balance principles, availability of soil nutrients, primarily nitrogen (e.g., BALANCE, BASFOR, Heterofor and iLand), can be used as a main driver for distributing C into tree compartments. The impact of drought can be simulated using an optimal partitioning theory since C allocation is dynamic with regard to the limiting source, e.g., in water limiting conditions more C is allocated to roots (Ostle et al. 2009, Pezzatti 2011). Farrior et al. (2013, 2015) applied an evolutionarily stable strategy to simulate the influence of water limitation on the C allocation of individual trees in a closed-canopy equilibrium forest. The most theoretically comprehensive approach from an evolutionary perspective is modelling on the base of adaptive dynamics (Franklin et al. 2012), which has however not been applied in any of the models studied here (Table 2).

Another approach on how to include direct environmental effects on C allocation in models is to modify allocation coefficients with regard to simulated resources, most commonly water (ANAFORE) and light (3D-CMCC FEM) or nitrogen (Xia et al. 2017) following, e.g., the work by Friedlingstein et al. (1999) or using dose–response curves for the responses of main plant fractions (i.e., leaf, stem and root) to environmental factors (Poorter et al. 2011). Drought disturbance effects on allocation are incorporated in models via altered respiration needs of each organ, altered order of preference for allocation, changed allocation ratios and/or applying the pipe model theory (Grote and Pretzsch 2002, Lasch et al. 2005, Van Oijen et al. 2005, Deckmyn et al. 2008, Rötzer et al. 2010, Jansson 2012). A model that includes a C allocation modifier, which responds to light, water availability or competition (e.g., 3D-CMCC FEM, ORCHIDEE-CAN) and is able to simulate particular disturbances, accounts for the impact of tree mortality triggered by windstorms, insect outbreaks or fire (e.g., iLand). Recently, frameworks on how to model insect and pathogen damage to affect the allocation, especially NSC, have been published (Dietze and Matthes 2014). An active role of NSC in C allocation (Martinez-Vilalta 2014) is considered in several models (e.g., 3D-CMCC FEM), which prioritize C allocation to reserves over biomass growth and use the reserve pool, e.g., for the production of leaves and fine roots at the beginning of the growing season.

Including seasonality in models of C allocation has been considered as a means of making the models capable of reflecting intra-annual environmental changes (Pretzsch 2009). At the sub-annual scale, growth and hence C allocation to different tissues varies following a seasonal pattern where the growth of different organs adheres to a species-specific sequence. For example, in oak species, cambial growth starts before the growth of foliage and primary wood, whereas in many conifers, it is the other way round (Michelot et al. 2012, Gričar et al. 2017, Schiestl-Aalto and Mäkelä 2017). The treatment of allocation can only be genuinely regarded as sub-annual if this seasonal rhythm is considered. The response of C allocation to various environmental factors incorporated using principles and/or types sensitive to environmental conditions (see Table 2) may be interpreted as a representation of seasonality in models. For, example a source–sink type of modelling C allocation implies that sink demand of all plant compartments changes dynamically throughout phenological stages (e.g., LANDSCAPE DNDC, CASTANEA, ANAFORE, CoupModel and 3D-CMCC FEM). Another option is to define seasons a priori using, e.g., a growing degree day threshold, which controls fruit formation (e.g., CLM-Palm and Biome-BGCMuSo). However, if such an approach is applied with allometric allocation, it should be regarded as a technical solution rather than trying to realistically mimic intra-annual C allocation patterns (‘average-day approximation’; Hastings and Gross 2012), since allometric relationships cannot be determined at a shorter time resolution than 1 year by any reasonable accuracy. During stand development, C allocation can be modified by implementing size-related allocation ratios, often based on the notion that different compartments try to maintain a particular balance (e.g., 3PG, ForGEM, CoupModel and ORCHIDEE-CAN).

To overcome the gap in considering reproduction, algorithms have been ‘borrowed’ from crop simulators (e.g., Pavlick et al. 2013). The onset and/or relative magnitude of allocation to fruits have been related to temperature, growing degree days, heat thresholds or day length (Oleson et al. 2013), and additional impacts of available water (Berg et al. 2010) and nitrogen (Hidy et al. 2016) have been considered. These models work for regularly fruiting trees or if only average allocation values throughout longer than annual time scales are required. Some examples also exist for introducing labile or NSC pools that distribute over other compartments in highly process-oriented forest growth models (Grote 1998, Deckmyn et al. 2008, Collalti et al. 2016). In resource budget models (Isagi et al. 1997, Crone and Rapp 2014), fruiting fluctuates from one year to the next when the tree produces seeds that subsequently deplete resource reserves. Pollination is considered as a limiting factor that may lead to fruiting failure and resource savings, which may be invested in flowering the following year (Satake and Iwasa 2000, Venner et al. 2016). In some models, flowering is inhibited in response to weather conditions of the same year (Abe et al. 2016).

Regarding other C pools considered for allocation, some specific approaches have been suggested that might be further elaborated or simplified. Models considering mycorrhiza have been reviewed by Deckmyn et al. (2014) and He et al. (2016), demonstrating the importance of considering plant–fungi feedback relations. An explicit dependence on root growth and soil nitrogen availability has been presented by Ruotsalainen et al. (2002) and Meyer et al. (2009, 2012). Moore et al. (2015) also included a dynamic switch of the role from plant symbiont to decomposer. Damage repair mechanisms have been considered in models describing the impact of air pollution (Van Oijen et al. 2005, Deckmyn et al. 2007), requiring a dynamic pool of C that might be linked to a general pool of free available C.

Data collection should aim for methods of direct quantification of C allocation enabling tracing of the path of C from the assimilation to formation of new structures. Sap flow measurements and labelling C isotopes appear to be promising methodologies for a better understanding of tree C dynamics (e.g., Kuptz et al. 2011, Klein et al. 2016a; McCarroll et al. 2017). Recent developments in tools to trace C isotopes, e.g., isotope ratio infrared spectroscopy, has contributed to a substantial increase in accuracy for the evaluation of C in ephemeral pools and transport rates, providing an important step towards a better understanding of C allocation processes (Epron et al. 2012*a*). For the evaluation of NSC, bomb radiocarbon measurements have been proposed (Carbone et al. 2013), as this method allows deriving the average time since the NSC was initially assimilated from the atmosphere (Hartmann and Trumbore 2016). When the use of allometric relationships is necessary, applying site and species-specific biomass measurements are warranted for evaluating and calibrating allocation models. Moreover, combining multiple data sources may overcome limitations on the temporal resolution required for the growth patterns of each C pool (Gea-Izquierdo et al. 2015). Luyssaert et al. (2007) collected results from multiple experiments describing C budget variables, ecosystem traits, management history and environmental variables, such as climate and soil characteristics. In a similar fashion, Bond-Lamberty and Thomson (2010) compiled a global dataset with soil respiration experiments, providing a basis for a better understanding of soil respiration dynamics, which usually require resource intensive experiments. Efforts for harmonizing and standardizing the datasets will be crucial for a better description of C allocation patterns.

## Discussion and conclusions

Since the first study about C allocation (Hartig 1878), this plant function has gained recognition both in experimental as well as in modelling studies, especially over the past 20 years (see Figure S4 available as Supplementary Data at *Tree Physiology* Online). This increasing attention results from ongoing climate change affecting the functioning of ecosystems both directly and indirectly (Charru et al. 2017). Based on our review and synthesis of experimental knowledge and modelling approaches, we suggest that the major challenge is to overcome key limitations in understanding of C allocation fundamentals, which can subsequently enhance its description in models, as already outlined by others (de Kauwe et al. 2014, Garcia et al. 2016).

### Challenges to fill the knowledge gaps in carbon allocation modelling

Despite considerable progress, a comprehensive picture of C allocation in trees is still missing. Improved empirical knowledge about C allocation in trees is of particular importance under changing environmental conditions because a realistic representation of processes in models may enhance their applicability in diverse situations (Seidl et al. 2011). There are several methodological issues to be solved, particularly those focusing on measuring carbohydrates in plant tissues and the accurate determination of their absolute concentrations (Quentin et al. 2015) and explaining the role of NSC in plant tissues (Carbone et al. 2013, Collalti et al. 2019*b*). The increased knowledge on the NSC accumulation and mobilization for metabolic activities would enhance not only our understanding of tree recovery and resilience adaptation mechanisms, but also the estimates of both aboveground and belowground NPP provided by models (Langley et al. 2002). The other areas of as yet limited scientific understanding in this field, which are likely to become more pressing issues with ongoing climate change, are the impact of disturbances on C allocation in trees, the production of seeds and fruit by trees and the C use by tree symbionts and/or for defense or repair. For a better understanding and mechanistic description of C allocation in models, more empirical studies dealing with these issues under changing environmental conditions are required (Guillemot et al. 2015, Sevanto and Dickman 2015).

### Carbon allocation modelling concepts in the view of climate change

Our analysis revealed that simpler empirical approaches of C allocation modelling prevail (Table 2), although they are not always able to capture the impact of environmental changes on C allocation. In general, dynamic C allocation schemes responsive to limiting factors aboveground and belowground should be favoured when modelling C allocation, because they can at least principally respond to new combinations of environmental conditions expected under climate change (Campioli et al. 2008). The most robust approach for modelling C allocation is a top-down evolutionary-based principle (Drewniak and Gonzalez-Meler 2017). Bottom-up approaches are apparently not able to capture complex allocation patterns controlled by the environment (Chen et al. 2013), although allocation schemes based on functional relationships and optimization theory are more robust than those based on fixed allocation or resource limitation principles (de Kauwe et al. 2014).

### Challenges for carbon allocation modelling under climate change

In the future, ensemble tests considering a number of modelling concepts on different spatial scales should be performed to find the principle that best meets observed responses as has been suggested by Cariboni et al. (2007) and Pianosi et al. (2016). Examples of such exercises can be found in Fischlin et al. (1995), Alvenäs and Jansson (1997), White et al. (2000), Pappas et al. (2013) and Montané et al. (2017). Such studies may clarify the effect of combining several principles/types of C allocation modelling, the approach that has been applied in the majority of investigated models (Figure 3). The results of ensemble simulations may also specify which parts of the model need improvements.

While there are several approaches for how to deal with the lack of sensitivity of C allocation models to environmental conditions either by using more sophisticated modelling principles or by implementing allocation modifiers (see Chapter Approaches and examples to overcome the gaps in carbon allocation modelling for more details), the impact of disturbances on C allocation cannot be simulated if the model does not account for them. Hence, the key step is to actually include disturbances and their impacts on forests in models (Seidl et al. 2011). Subsequently, the disturbances can be linked to the processes governing C allocation in models, while experimental studies should be used as a platform for model development.

Implementing new features in the model to improve simulated C allocation processes should be performed with regard to the research question and study location. Including specific nutrient dynamics in models may be important for future projections of the C cycle in regions where the particular nutrient is limited (Zaehle 2013). In nitrogen-limited forests, implementing nitrogen dynamics and/or C allocation to symbionts significantly enhance the predictive power of models (de Kauwe et al. 2014, Wårlind et al. 2014, He et al. 2018). In the future, other nutrients may constrain forest productivity. The modelling approach of phosphorus cycling in ecosystems implemented in ANAFORE may serve as an example how to account for the effects of its deficiency on C allocation (Bortier et al. 2018).

From the viewpoint of long-term plant strategy, successful reproduction is a major evolutionary goal of C allocation (Agren and Wikstrom 1993). Hence, omitting to allocate C into reproductive organs, particularly during masting years, may be a cause of low prediction accuracy of forest models (Vacchiano et al. 2018). If variable allocation across years is aimed for, then the NSC dynamics need to be included in the allocation pattern, and the interaction of inter- and intra-annual C allocation must be considered. The size of the NSC pool might be used to define feedback to photosynthesis, thus decreasing the atmospheric CO_2_ effect. The question of time steps is crucial when defining the role of C in stress responses and tree mortality, where the availabilty of reserves may be the decisive determinant of survival (Dietze et al. 2014). By uncoupling photosynthesis and growth under stress conditions, e.g., drought, a more realistic representation of the carry-over effect of stress periods on growth can be obtained due to buffering power of the C storage/reserve pool. This pool needs to be dynamic and may change size based on short-term stress occurrence (induced defences) or long-term stress intensity (acclimation) (Hartmann and Trumbore 2016).

We conclude that to obtain reliable output from models under climate change, modellers should consider: (i) using more sensitive to changing environmental conditions C allocation modelling approaches (Table 2); (ii) integrating factors and processes the importance of which is assumed to increase in the future, such as disturbances, reproduction, symbiosis, defence and repair; and (iii) incorporating currently underrepresented C pools for NSCs, fruit and seed production, and mycorrhiza. Although some approaches are already available and have been synthesized in Chapter Approaches and examples to overcome the gaps in carbon allocation modelling, more comprehensive understanding of the shifts in C allocation due to changes in environment, which can be obtained from controlled or manipulative experiments, may further improve its representation in models. Yet, any model improvements need to be performed to balance the trade-off between model complexity and model robustness; conceptually sound, experimentally supported processes that are consistent with the general model structure need to be pursued.

## Supplementary Material

Merganicova_etal_CarbonAllocation_17092019_Suplement_tpz105

## References

[1] Abe T, Tachiki Y, Kon H, Nagasaka A, Onodera K, Minamino K, Han Q, Satake A (2016) Parameterisation and validation of a resource budget model for masting using spatiotemporal flowering data of individual trees. Ecol Lett 19:1129–1139. 27449602 10.1111/ele.12651

[2] Aber JD, Federer CA (1992) A generalized, lumped-parameter model of photosynthesis, evapotranspiration and net primary production in temperate and boreal forest ecosystems. Oecologia 92:463–474. 28313216 10.1007/BF00317837

[3] Agren GI, Wikstrom JF (1993) Modelling carbon allocation—a review. N Z J For Sci 23:343–353.

[4] Allister KM, Harding LA, Vernon Cole C, Parton WJ (1993) CENTURY Soil Organic Matter Model Environment. https://www2.nrel.colostate.edu/projects/century/MANUAL/html_manual/man96.html. (June 2, 2019, date last accessed).

[5] Alvenäs G, Jansson P-E (1997) Model for evaporation, moisture and temperature of bare soil: calibration and sensitivity analysis. Agric For Meteorol 88:47–56.

[6] Anić M, Ostrogović Sever MZ, Alberti G et al. (2018) Eddy covariance vs. biometric based estimates of net primary productivity of pedunculate oak (*Quercus robur* L.) forest in Croatia during ten years. Forests 9:764.

[7] Arora VK, Boer GJ (2005) A parameterization of leaf phenology for the terrestrial ecosystem component of climate models. Glob Chang Biol 11:39–59.

[8] Ascoli D, Maringer J, Hacket-Pain A et al. (2017) Two centuries of masting data for European beech and Norway spruce across the European continent. Ecology 98:1473–1473. 28241388 10.1002/ecy.1785

[9] Bansal S, Hallsby G, Lofvenius MO, Nilsson M-C (2013) Synergistic, additive and antagonistic impacts of drought and herbivory on *Pinus sylvestris*: leaf, tissue and whole-plant responses and recovery. Tree Physiol 33:451–463. 23525156 10.1093/treephys/tpt019

[10] Barigah TS, Bonhomme M, Lopez D, Traore A, Douris M, Venisse J-S, Cochard H, Badel E (2013) Modulation of bud survival in *Populus nigra* sprouts in response to water stress-induced embolism. Tree Physiol 33:261–274. 23467748 10.1093/treephys/tpt002

[11] Bellgard SE, Williams SE (2011) Response of mycorrhizal diversity to current climatic changes. Diversity 3:8–90.

[12] Berg A, Sultan B, de Noblet-Ducoudré N (2010) Including tropical croplands in a terrestrial biosphere model: application to West Africa. Clim Change 104:755–782.

[13] Bohn FJ, Frank K, Huth A (2014) Of climate and its resulting tree growth: simulating the productivity of temperate forests. Ecol Model 278:9–17.

[14] Bond-Lamberty B, Thomson A (2010) Temperature-associated increases in the global soil respiration record. Nature 464:579–582. 20336143 10.1038/nature08930

[15] Bortier MF, Andivia E, Genon JG, Grebenc T, Deckmyn G (2018) Towards understanding the role of ectomycorrhizal fungi in forest phosphorus cycling: a modelling approach. Cent Eur For J 64:79–95.

[17] Bugmann H, Grote R, Lasch P, Lindner M, Suckow F (1997) A new forest gap model to study the effects of environmental change on forest structure and functioning In: Mohren GMJ, Kramer K, Sabatè S (eds.) Impacts of Global Change on Tree Physiology and Forest Ecosystems. Forestry Sciences vol 52 Dordrecht: Springer, 255–261. 10.1007/978-94-015-8949-9_33. (8 December 2018, date last accessed)

[18] Bugmann HKM (1994) On the ecology of mountainous forests in a changing climate: a simulation study. Doctoral thesis, ETH Zurich. https://www.research-collection.ethz.ch/handle/20.500.11850/141625. (7 December 2018, date last accessed).

[19] Caldararu S, Purves DW, Palmer PI (2014) Phenology as a strategy for carbon optimality: a global model. Biogeosciences 11:763–778.

[21] Campioli M, Verbeeck H, Lemeur R, Samson R (2008) C allocation among fine roots, above-, and belowground wood in a deciduous forest and its implication to ecosystem C cycling: a modelling analysis. Biogeosciences 5:3781–3823.

[20] Campioli M, Malhi Y, Vicca S et al (2016) Evaluating the convergence between eddy-covariance and biometric methods for assessing carbon budgets of forests. Nat Commun 7:13717. 27966534 10.1038/ncomms13717 PMC5171944

[22] Cannell MGR, Dewar RC (1994) Carbon allocation in trees: a review of concepts for modelling. Academic Press, London, San Diego.

[23] Carbone MS, Czimczik CI, Keenan TF, Murakami PF, Pederson N, Schaberg PG, Xu X, Richardson AD (2013) Age, allocation and availability of nonstructural carbon in mature red maple trees. New Phytol 200:1145–1155. 24032647 10.1111/nph.12448

[24] Cariboni J, Gatelli D, Liska R, Saltelli A (2007) The role of sensitivity analysis in ecological modelling. Ecol Model 203:167–182.

[25] Charru M, Seynave I, Hervé J-C, Bertrand R, Bontemps J-D (2017) Recent growth changes in Western European forests are driven by climate warming and structured across tree species climatic habitats. Ann For Sci 74:33.

[26] Chen G, Yang Y, Robinson D (2013) Allocation of gross primary production in forest ecosystems: allometric constraints and environmental responses. New Phytol 200:1176–1186. 23902539 10.1111/nph.12426

[ref26a] Cleland EE, Chuine I, Menzel A, Mooney HA, Schwartz MD (2013) Shifting plant phenology in response to global change. Trends in Ecology & Evolution. 22: 357–365. 10.1016/j.tree.2007.04.003. 17478009

[29] Collalti A, Prentice IC (2019) Is NPP proportional to GPP? Waring’s hypothesis twenty years on. Tree Physiol. 39:1473–1483. doi: 10.1093/treephys/tpz0.34, (2 June 2019, date last accessed). 30924876

[28] Collalti A, Perugini L, Santini M, Chiti T, Nolè A, Matteucci G, Valentini R (2014) A process-based model to simulate growth in forests with complex structure: evaluation and use of 3D-CMCC forest ecosystem model in a deciduous forest in Central Italy. Ecol Model 272:362–378.

[27] Collalti A, Marconi S, Ibrom A et al. (2016) Validation of 3D-CMCC forest ecosystem model (v.5.1) against eddy covariance data for 10 European forest sites. Geosci Model Dev 9:479–504.

[32] Collalti A, Trotta C, Keenan TF et al. (2018) Thinning can reduce losses in carbon use efficiency and carbon stocks in managed forests under warmer climate. J Adv Modeling Earth Systems 10:2427–2452. 10.1029/2018ms001275. (2 June 2019, date last accessed).. PMC6472666 31007835

[30] Collalti A, Thornton PE, Cescatti A, Rita A, Borghetti M, Nolè A, Trotta C, Ciais P, Matteucci G (2019 *a*) The sensitivity of the forest carbon budget shifts across processes along with stand development and climate change. Ecol Appl 29:e01837. 30549378 10.1002/eap.1837 PMC6849766

[31] Collalti A, Tjoelker MG, Hoch G et al. (2019 *b*) Plant respiration: controlled by photosynthesis or biomass? Glob Chang Biol. doi: 10.1101/705400. 31578796

[33] Courty PE, Buée M, Diedhiou AG, Frey-Klett P, Le Tacon F, Rineau F et al. (2010) The role of ectomycorrhizal communities in forest ecosystem processes: new perspectives and emerging concepts. Soil Biol Biochem 42:679–698.

[34] Coutand C, Dupraz C, Jaouen G, Ploquin S, Adam B (2008) Mechanical stimuli regulate the allocation of biomass in trees: demonstration with young *Prunus avium* trees. Ann Bot 101:1421–1432. 18448448 10.1093/aob/mcn054 PMC2710262

[35] Crone EE, Rapp JM (2014) Resource depletion, pollen coupling, and the ecology of mast seeding. Ann N Y Acad Sci 1322:21–34. 24888210 10.1111/nyas.12465

[36] Da Silva D, Favreau R, Auzmendi I, DeJong TM (2011) Linking water stress effects on carbon partitioning by introducing a xylem circuit into L-PEACH. Ann Bot 108:1135–1145. 21546432 10.1093/aob/mcr072 PMC3189834

[113] de Kauwe MG, Medlyn BE, Zaehle S et al. (2014) Where does the carbon go? A model-data intercomparison of vegetation carbon allocation and turnover processes at two temperate forest free-air CO2 enrichment sites. New Phytol 203:883–899. 24844873 10.1111/nph.12847 PMC4260117

[260] de Willigen P (1991) Nitrogen turnover in the soil-crop system; comparison of fourteen simulation models. Fert Res 27:141–149.

[38] Deckmyn G, Op de Beeck M, Löw M, Then C, Verbeeck H, Wipfler P, Ceulemans R (2007) Modelling ozone effects on adult beech trees through simulation of defence, damage, and repair costs: implementation of the CASIROZ ozone model in the ANAFORE forest model. Plant Biol 9:320–330. 17357024 10.1055/s-2006-924762

[37] Deckmyn G, Meyer A, Smits MM, Ekblad A, Grebenc T, Komarov A, Kraigher H (2014) Simulating ectomycorrhizal fungi and their role in carbon and nitrogen cycling in forest ecosystems. Can J For Res 44:535–553.

[39] Deckmyn G, Verbeeck H, Op de Beeck M, Vansteenkiste D, Steppe K, Ceulemans R (2008) ANAFORE: a stand-scale process-based forest model that includes wood tissue development and labile carbon storage in trees. Ecol Model 215:345–368.

[40] Delpierre N, Vitasse Y, Chuine I, Guillemot J, Bazot S, Rutishauser T, Rathgeber CBK (2015) Temperate and boreal forest tree phenology: from organ-scale processes to terrestrial ecosystem models. Ann For Sci 73:5–25.

[41] DeLucia EH, Maherali H, Carey EV (2000) Climate-driven changes in biomass allocation in pines. Glob Chang Biol 6:587–593.

[42] Dietze MC, Matthes JH (2014) A general ecophysiological framework for modelling the impact of pests and pathogens on forest ecosystems. Ecol Lett 17:1418–1426. 25168168 10.1111/ele.12345 PMC4257091

[43] Dietze MC, Sala A, Carbone MS, Czimczik CI, Mantooth JA, Richardson AD, Vargas R (2014) Nonstructural carbon in Woody plants. Annu Rev Plant Biol 65:667–687. 24274032 10.1146/annurev-arplant-050213-040054

[44] Drewniak B, Gonzalez-Meler M (2017) Earth system model needs for including the interactive representation of nitrogen deposition and drought effects on forested ecosystems. Forests 8:267.

[45] Drobyshev I, Övergaard R, Saygin I, Niklasson M, Hickler T, Karlsson M, Sykes MT (2010) Masting behaviour and dendrochronology of European beech (*Fagus sylvatica* L.) in southern Sweden. For Ecol Manage 259:2160–2171.

[46] Drouet J-L, Pagès L (2007) GRAAL-CN: a model of GRowth, architecture and ALlocation for carbon and nitrogen dynamics within whole plants formalised at the organ level. Ecol Model 206:231–249.

[47] Dufrêne E, Davi H, François C, Maire G, leDantec VL, Granier A (2005) Modelling carbon and water cycles in a beech forest. Ecol Model 185:407–436.

[48] Eckersten H, Jansson P-E (1991) Modelling water flow, nitrogen uptake and production for wheat. Fert Res 27:313–329.

[49] Eis S, Garman EH, Ebell LF (1965) Relation between cone production and diameter increment of Douglas fir (*Pseudotsuga menziesii* (Mirb.) Franco), grand fir (*Abies grandis* (Dougl.) Lindl.), and Western white pine (*Pinus monticola* Dougl.). Can J Bot 43:1553–1559.

[50] Ekblad A, Wallander H, Carlsson R, Huss-Danell K (1995) Fungal biomass in roots and extramatrical mycelium in relation to macronutrients and plant biomass of ectomycorrhizal *Pinus sylvestris* and *Alnus incana*. New Phytol 131:443–451. 10.1111/j.1469-8137.1995.tb03081.x 33863123

[51] Endrulat T, Buchmann N, Brunner I (2016) Carbon allocation into different fine-root classes of young *Abies alba* trees is affected more by phenology than by simulated browsing. PLoS One 11:e0154687. 27123860 10.1371/journal.pone.0154687 PMC4849635

[53] Epron D, Laclau J-P, Almeida JCR, Goncalves JLM, Ponton S, Sette CR, Delgado-Rojas JS, Bouillet J-P, Nouvellon Y (2011) Do changes in carbon allocation account for the growth response to potassium and sodium applications in tropical eucalyptus plantations? Tree Physiol 32:667–679. 22021011 10.1093/treephys/tpr107

[52] Epron D, Bahn M, Derrien D et al. (2012 *a*) Pulse-labelling trees to study carbon allocation dynamics: a review of methods, current knowledge and future prospects. Tree Physiol 32:776–798. 22700544 10.1093/treephys/tps057

[54] Epron D, Nouvellon Y, Ryan MG (2012 *b*) Introduction to the invited issue on carbon allocation of trees and forests. Tree Physiol 32:639–643. 22730478 10.1093/treephys/tps055

[55] Ericsson T (1995) Growth and shoot: root ratio of seedlings in relation to nutrient availability In: Nilsson LO, Hüttl RF, Johansson UT (eds.) Nutrient Uptake and Cycling in Forest Ecosystems vol. 62 Dordrecht: Springer, 205–214. 10.1007/978-94-011-0455-5_23 (8 December 2018, date last accessed).

[56] Eyles A, Pinkard EA, Mohammed C (2009) Shifts in biomass and resource allocation patterns following defoliation in *Eucalyptus globulus* growing with varying water and nutrient supplies. Tree Physiol 29:753–764. 19324694 10.1093/treephys/tpp014

[57] Eziz A, Yan Z, Tian D, Han W, Tang Z, Fang J (2017) Drought effect on plant biomass allocation: a meta-analysis. Ecol Evol 7:11002–11010. 29299276 10.1002/ece3.3630 PMC5743700

[58] Fabrika M (2005) Forest biodynamic simulator SIBYLA, conception, construction and program solution. PhD thesis, Technical University in Zvolen.

[59] Fabrika M, Ďurský J (2006) Implementing tree growth models in Slovakia In: Hasenauer H (ed) Sustainable forest management: growth models for Europe. Springer, Berlin, Heidelberg, pp 315–341. 10.1007/3-540-31304-4_19. (8 December 2018, date last accessed).

[60] Fabrika M, Pretzsch H (2011) Analýza a modelovanie lesných ekosystémov. Technická univerzita vo Zvolene.

[61] Fan Y, Roupsard O, Bernoux M, Le Maire G, Panferov O, Kotowska MM, Knohl A (2015) A sub-canopy structure for simulating oil palm in the community land model (CLM-Palm): phenology, allocation and yield. Geosci Model Dev 8:3785–3800.

[62] Farrior CE, Dybzinski R, Levin SA, Pacala SW (2013) Competition for water and light in closed-canopy forests: a tractable model of carbon allocation with implications for carbon sinks. Am Nat 181:314–330. 23448882 10.1086/669153

[63] Farrior CE, Rodriguez-Iturbe I, Dybzinski R, Levin SA, Pacala SW (2015) Decreased water limitation under elevated CO2 amplifies potential for forest carbon sinks. Proc Natl Acad Sci USA 112:7213–7218. 26039985 10.1073/pnas.1506262112 PMC4466696

[64] Fatichi S, Leuzinger S (2013) Reconciling observations with modeling: the fate of water and carbon allocation in a mature deciduous forest exposed to elevated CO2. Agric For Meteorol 174–175:144–157.

[65] Fatichi S, Leuzinger S, Körner C (2014) Moving beyond photosynthesis: from carbon source to sink-driven vegetation modeling. New Phytol 201:1086–1095. 24261587 10.1111/nph.12614

[66] Ferrieri AP, Agtuca B, Appel HM, Ferrieri RA, Schultz JC (2013) Temporal changes in allocation and partitioning of new carbon as 11C elicited by simulated Herbivory suggest that roots shape aboveground responses in *Arabidopsis*. Plant Physiol 161:692–704. 23370716 10.1104/pp.112.208868 PMC3561013

[67] Fischlin A, Bugmann H, Gyalistras D (1995) Sensitivity of a forest ecosystem model to climate parameterization schemes. Environ Pollut 87:267–282. 15091576 10.1016/0269-7491(94)p4158-k

[68] Fontes L, Bontemps J-D, Bugmann H, Oijen MV, Gracia C, Kramer K, Lindner M, Rötzer T, Skovsgaard JP (2010) Models for supporting forest management in a changing environment. Forest Syst 19:8–29.

[69] Franklin O, Johansson J, Dewar RC, Dieckmann U, McMurtrie RE, Brannstrom A, Dybzinski R (2012) Modeling carbon allocation in trees: a search for principles. Tree Physiol 32:648–666. 22278378 10.1093/treephys/tpr138

[70] Friedlingstein P, Joel G, Field CB, Fung IY (1999) Toward an allocation scheme for global terrestrial carbon models. Glob Chang Biol 5:755–770.

[71] Friend AD, Lucht W, Rademacher TT et al. (2013) Carbon residence time dominates uncertainty in terrestrial vegetation responses to future climate and atmospheric CO2. Proc Natl Acad Sci USA 111:3280–3285. 24344265 10.1073/pnas.1222477110 PMC3948236

[72] Galvez DA, Landhausser SM, Tyree MT (2011) Root carbon reserve dynamics in aspen seedlings: does simulated drought induce reserve limitation? Tree Physiol 31:250–257. 21444372 10.1093/treephys/tpr012

[73] Garcia ES, Tague CL, Choate JS (2016) Uncertainty in carbon allocation strategy and ecophysiological parameterization influences on carbon and streamflow estimates for two western US forested watersheds. Ecol Model 342:19–33.

[74] Gayler S, Grams TEE, Heller W, Treutter D, Priesack E (2007) A dynamical model of environmental effects on allocation to carbon-based secondary compounds in juvenile trees. Ann Bot 101:1089–1098. 17693454 10.1093/aob/mcm169 PMC2710266

[75] Gea-Izquierdo G, Guibal F, Joffre R, Ourcival JM, Simioni G, Guiot J (2015) Modelling the climatic drivers determining photosynthesis and carbon allocation in evergreen Mediterranean forests using multiproxy long time series. Biogeosciences 12:3695–3712.

[76] Genet H, Breda N, Dufrene E (2009) Age-related variation in carbon allocation at tree and stand scales in beech (*Fagus sylvatica* L.) and sessile oak (*Quercus petraea* (Matt.) Liebl.) using a chronosequence approach. Tree Physiol 30:177–192. 20018984 10.1093/treephys/tpp105

[78] Gough CM, Vogel CS, Schmid HP, Su H-B, Curtis PS (2008) Multi-year convergence of biometric and meteorological estimates of forest carbon storage. Agric For Meteorol 148:158–170.

[77] Gough CM, Flower CE, Vogel CS, Dragoni D, Curtis PS (2009) Whole-ecosystem labile carbon production in a north temperate deciduous forest. Agric For Meteorol 149:1531–1540.

[79] Gričar J, Lavrič M, Ferlan M, Vodnik D, Eler K (2017) Intra-annual leaf phenology, radial growth and structure of xylem and phloem in different tree parts of *Quercus pubescens*. Eur J For Res 136:625–637.

[80] Grote R (1998) Integrating dynamic morphological properties into forest growth modelling. For Ecol Manage 111:193–210.

[82] Grote R, Pretzsch H (2002) A model for individual tree development based on physiological processes. Plant Biol 4:167–180.

[83] Grote R, Reiter IM (2004) Competition-dependent modelling of foliage biomass in forest stands. Trees 18: 596–607. https://www.academia.edu/19549960/Competition-dependent_modelling_of_foliage_biomass_in_forest_stands. (2 June 2019, date last accessed).

[81] Grote R, Kiese R, Grünwald T, Ourcival J-M, Granier A (2011) Modelling forest carbon balances considering tree mortality and removal. Agric For Meteorol 151:179–190.

[85] Guillemot J, Martin-StPaul NK, Dufrêne E, François C, Soudani K, Ourcival JM, Delpierre N (2015) The dynamic of the annual carbon allocation to wood in European tree species is consistent with a combined source–sink limitation of growth: implications for modelling. Biogeosciences 12:2773–2790.

[84] Guillemot J, Francois C, Hmimina G, Dufrêne E, Martin-StPaul NK, Soudani K, Marie G, Ourcival J-M, Delpierre N (2016) Environmental control of carbon allocation matters for modelling forest growth. New Phytol 214:180–193. 27883190 10.1111/nph.14320

[86] Gutiérrez AG, Armesto JJ, Díaz MF, Huth A (2014) Increased drought impacts on temperate rainforests from southern South America: results of a process-based dynamic forest model. PLoS One 9:e103226. 25068869 10.1371/journal.pone.0103226 PMC4113359

[87] Hacket-Pain AJ, Friend AD, Lageard JGA, Thomas PA (2015) The influence of masting phenomenon on growth-climate relationships in trees: explaining the influence of previous summers’ climate on ring width. Tree Physiol 35:319–330. 25721369 10.1093/treephys/tpv007

[88] Hagedorn F, Joseph J, Peter M et al. (2016) Recovery of trees from drought depends on belowground sink control. Nat Plants 2: 16111 (article number), 5pp. (1-5) 10.1038/nplants.2016.111. (6 December 2018, date last accessed). 27428669

[89] Halman JM, Schaberg PG, Hawley GJ, Pardo LH, Fahey TJ (2013) Calcium and aluminum impacts on sugar maple physiology in a northern hardwood forest. Tree Physiol 33:1242–1251. 24300338 10.1093/treephys/tpt099

[90] Hartig R (1878) Die zersetzungserscheinungen des holzes der nadelholzbäume und der eiche in forstlicher, botanischer und chemischer richtung J. Berlin: Springer.

[91] Hartmann H (2015) Carbon starvation during drought-induced tree mortality – are we chasing a myth? J Plant Hydraulics 2:005.

[92] Hartmann H, Trumbore S (2016) Understanding the roles of nonstructural carbohydrates in forest trees - from what we can measure to what we want to know. New Phytol 211:386–403. 27061438 10.1111/nph.13955

[93] Hasselquist NJ, Metcalfe DB, Inselsbacher E, Stangl Z, Oren R, Näsholm T, Högberg P (2016) Greater carbon allocation to mycorrhizal fungi reduces tree nitrogen uptake in a boreal forest. Ecology. 97: 1012–1022. 10.1890/15-1222 (5 December 2018, date last accessed). 27220217

[94] Hastings A, Gross LJ (2012) Encyclopedia of theoretical ecology, 1st edn. University of California Press, Oakland, CA, USA, http://www.jstor.org/stable/10.1525/j.ctt1pp0s7. (8 December 2018, date last accessed).

[95] He H, Jansson P-E, Svensson M, Björklund J, Tarvainen L, Klemedtsson L, Kasimir Å (2016) Forests on drained agricultural peatland are potentially large sources of greenhouse gases – insights from a full rotation period simulation. Biogeosciences 13:2305–2318.

[96] He H, Meyer A, Jansson P-E, Svensson M, Rütting T, Klemedtsson L (2018) Simulating ectomycorrhiza in boreal forests: implementing ectomycorrhizal fungi model MYCOFON in CoupModel (v5). Geosci Model Dev 11:725–751.

[98] Hicke JA, Allen DC, Desai AR et al. (2012) Effects of biotic disturbances on forest carbon cycling in the United States and Canada. Glob Chang Biol 18:7–34.

[99] Hidy D, Barcza Z, Marjanović H et al. (2016) Terrestrial ecosystem process model biome-BGCMuSo: summary of improvements and new modeling possibilities. Geosci Model Dev 1–60.

[100] Hobbie EA (2006) Carbon allocation to ectomycorrhizal fungi correlates with belowground allocation in culture studies. Ecology 87:563–569. 16602286 10.1890/05-0755

[101] Hoch G, Richter A, Korner C (2003) Non-structural carbon compounds in temperate forest trees. Plant Cell Environ 26:1067–1081.

[102] Hoch G, Siegwolf RTW, Keel SG, Körner C, Han Q (2013) Fruit production in three masting tree species does not rely on stored carbon reserves. Oecologia 171:653–662. 23306421 10.1007/s00442-012-2579-2

[103] Holmsgaard E (1955) Tree-ring analyses of Danish forest trees. Tree-Ring Bulletin 22: pp 76–96, http://hdl.handle.net/10150/258968. (8 December 2018, date last accessed).

[104] Hommel R, Siegwolf R, Zavadlav S, Arend M, Schaub M, Galiano L, Haeni M, Kayler ZE, Gessler A (2016) Impact of interspecific competition and drought on the allocation of new assimilates in trees. Plant Biol 18:785–796. 27061772 10.1111/plb.12461

[105] Hurtt GC, Moorcroft PR, Pacala SW (2013) Ecosystem demography model: scaling vegetation dynamics across South America ORNL DAAC, Oak Ridge, Tennessee, USA. 10.3334/ORNLDAAC/1149 (2 June 2019, date last accessed).

[106] Isagi Y, Sugimura K, Sumida A, Ito H (1997) How does masting happen and synchronize? J Theor Biol 187:231–239.

[107] Jacquet J-S, Orazio C, Jactel H (2012) Defoliation by processionary moth significantly reduces tree growth: a quantitative review. Ann For Sci 69:857–866.

[108] Jansson P-E (2012) CoupModel: model use, calibration, and validation. Trans ASABE 55:1337–1346.

[109] Jansson P-E, Karlberg L (2004) Coupled heat and mass transfer model for soil-plant-atmosphere systems. Royal Institute of Technology, Department of Civil and Environmental Engineering, Stockholm, 321 pp., ftp://www.lwr.kth.se/CoupModel/CoupModel.pdf, (8 December 2018, date last accessed).

[110] Jin W, He HS, Thompson FR (2016) Are more complex physiological models of forest ecosystems better choices for plot and regional predictions? Environ Model Software 75:1–14.

[111] Jonard M, André F (2018) Heterofor [Capsis]. http://capsis.cirad.fr/capsis/help_en/heterofor. (8 December 2018, date last accessed).

[112] Kasurinen A, Biasi C, Holopainen T, Rousi M, Maenpaa M, Oksanen E (2012) Interactive effects of elevated ozone and temperature on carbon allocation of silver birch (*Betula pendula*) genotypes in an open-air field exposure. Tree Physiol 32:737–751. 22363070 10.1093/treephys/tps005

[114] Keenan T, Niinemets Ü, Sabate S, Gracia C, Peñuelas J (2009) Process based inventory of isoprenoid emissions from European forests: model comparisons, current knowledge and uncertainties. Atmos Chem Phys 9:4053–4076.

[115] Klein T, Hoch G (2014) Tree carbon allocation dynamics determined using a carbon mass balance approach. New Phytol 205:147–159. 25157793 10.1111/nph.12993

[116] Klein T, Siegwolf RTW, Korner C (2016 *a*) Belowground carbon trade among tall trees in a temperate forest. Science 352:342–344. 27081070 10.1126/science.aad6188

[118] Konôpka B, Lukac M (2012) Moderate drought alters biomass and depth distribution of fine roots in Norway spruce. For Pathol 43:115–123.

[119] Körner C (2003) Carbon limitation in trees. J Ecol 91:4–17.

[120] Körner C (2015) Paradigm shift in plant growth control. Curr Opin Plant Biol 25:107–114. 26037389 10.1016/j.pbi.2015.05.003

[121] Kozlowski TT (1992) Carbohydrate sources and sinks in woody plants. Bot Rev 58:107–222.

[124] Kramer K, van der Werf DC (2010) Equilibrium and non-equilibrium concepts in forest genetic modelling: population- and individually-based approaches. Forest Syst 19:100–112.

[122] Kramer K, Buiteveld J, Forstreuter M et al. (2008) Bridging the gap between ecophysiological and genetic knowledge to assess the adaptive potential of European beech. Ecol Model 216:333–353.

[123] Kramer K, van der Werf B, Schelhaas M-J (2015) Bring in the genes: genetic-ecophysiological modeling of the adaptive response of trees to environmental change. With application to the annual cycle. Front Plant Sci 5: 1–10 10.3389/fpls.2014.00742. (8 December 2018, date last accessed).. PMC4292233 25628628

[125] Krejza J, Pokorný R, Marková I (2013) Is allometry for aboveground organ’s mass estimation in young Norway spruce stands affected by different type of thinning? Acta Univ Agric et Silvic Mendel Brun 61:1755–1761.

[126] Kuptz D, Fleischmann F, Matyssek R, Grams TEE (2011) Seasonal patterns of carbon allocation to respiratory pools in 60-yr-old deciduous (*Fagus sylvatica*) and evergreen (*Picea abies*) trees assessed via whole-tree stable carbon isotope labeling. New Phytol 191:160–172. 21395596 10.1111/j.1469-8137.2011.03676.x

[127] Lacointe A (2000) Carbon allocation among tree organs: a review of basic processes and representation in functional-structural tree models. Ann For Sci 57:521–533.

[128] Landsberg JJ, Waring RH (1997) A generalised model of forest productivity using simplified concepts of radiation-use efficiency, carbon balance and partitioning. For Ecol Manage 95:209–228.

[129] Langley J, Drake B, Hungate B (2002) Extensive belowground carbon storage supports roots and mycorrhizae in regenerating scrub oaks. Oecologia 131:542–548. 28547549 10.1007/s00442-002-0932-6

[130] Lasch F, Le Roy F, Yami S (2005) Les déterminants de la croissance des start-up TIC. Rev Fr Gest 31:37–56.

[131] Lasch-Born P, Suckow F, Reyer COP et al. (2019) Description and evaluation of the process-based forest model 4C at four European forest sites. Geosci Model Dev 1–42. 10.5194/gmd-2019-2, in review

[132] Le Roux X, Lacointe A, Escobar-Gutierrez A, Le Dizes S (2001) Carbon-based models of individual tree growth: a critical appraisal. Ann For Sci 58:469–506.

[133] Lehtonen A, Heikkinen J (2015) Uncertainty of upland soil carbon sink estimate for Finland. Can J For Res 46:310–322.

[134] Lexer MJ, Hönninger K (2001) A modified 3D-patch model for spatially explicit simulation of vegetation composition in heterogeneous landscapes. For Ecol Manage 144:43–65.

[135] Li G, Harrison SP, Prentice IC (2016) A model analysis of climate and CO_2_ controls on tree growth and carbon allocation in a semi-arid woodland. Ecol Model 342:175–185.

[136] Lischke H, Zimmermann NE, Bolliger J, Rickebusch S, Löffler TJ (2006) TreeMig: a forest-landscape model for simulating spatio-temporal patterns from stand to landscape scale. Ecol Model 199:409–420.

[137] Litton CM, Giardina CP (2008) Below-ground carbon flux and partitioning: global patterns and response to temperature. Funct Ecol 22:941–954.

[138] Litton CM, Raich JW, Ryan MG (2007) Carbon allocation in forest ecosystems. Glob Chang Biol 13:2089–2109.

[139] Liu J-F, Arend M, Yang W-J, Schaub M, Ni Y-Y, Gessler A, Jiang Z-P, Rigling A, Li M-H (2017) Effects of drought on leaf carbon source and growth of European beech are modulated by soil type. Sci Rep 7: 1–10. 10.1038/srep42462. (8 December 2018, date last accessed). 28195166 PMC5307967

[140] Lonsdale J, Xenakis G, Mencuccini M, Perks M (2015) A comparison of models for quantifying growth and standing carbon in UK scots pine forests. iForest 8:596–605.

[141] Loustau D (2010) Forests, carbon cycle and climate change. Inra, Versailles Cedex: Editions Quae.

[142] Loustau D, Bosc A, Colin A et al. (2005) Modeling climate change effects on the potential production of French plains forests at the sub-regional level. Tree Physiol 25:813–823. 15870051 10.1093/treephys/25.7.813

[143] Lüdeke MKB, Badeck F-W, Otto RD et al. (1994) The Frankfurt biosphere model: a global process-oriented model of seasonal and long-term CO2 exchange between terrestrial ecosystems and the atmosphere. I. Model description and illustrative results for cold deciduous and boreal forests. Clim Res 4:143–166.

[144] Luyssaert S, Inglima I, Jung M et al. (2007) CO_2_ balance of boreal, temperate, and tropical forests derived from a global database. Glob Chang Biol 13:2509–2537.

[145] Mäkelä A (2012) On guiding principles for carbon allocation in eco-physiological growth models. Tree Physiol 32:644–647. 22730479 10.1093/treephys/tps033

[146] Mäkelä A, Landsberg J, Ek AR, Burk TE, Ter-Mikaelian M, Ågren GI, Oliver CD, Puttonen P (2000) Process-based models for forest ecosystem management: current state of the art and challenges for practical implementation. Tree Physiol 20:289–298. 12651445 10.1093/treephys/20.5-6.289

[147] Mäkelä A, Pulkkinen M, Mäkinen H (2016) Bridging empirical and carbon-balance based forest site productivity – significance of below-ground allocation. For Ecol Manage 372:64–77.

[148] Marconi S, Chiti T, Nolè A, Valentini R, Collalti A (2017) The role of respiration in estimation of net carbon cycle: coupling soil carbon dynamics and canopy turnover in a novel version of 3D-CMCC forest ecosystem model. Forests 8:220.

[149] Martín D, Vázquez-Piqué J, Carevic FS, Fernández M, Alejano R (2015) Trade-off between stem growth and acorn production in holm oak. Trees 29:825–834.

[150] Martinez-Vilalta J (2014) Carbon storage in trees: pathogens have their say. Tree Physiol 34:215–217. 24619516 10.1093/treephys/tpu010

[151] Mayfield AE III, Allen DC, Briggs RD (2005) Radial growth impact of pine false webworm defoliation on eastern white pine. Can J For Res 35:1071–1086.

[152] McCarroll D, Whitney M, Young GHF, Loader NJ, Gagen MH (2017) A simple stable carbon isotope method for investigating changes in the use of recent versus old carbon in oak. Tree Physiol 37:1021–1027. 28338989 10.1093/treephys/tpx030

[153] McDowell NG (2011) Mechanisms linking drought, hydraulics, carbon metabolism, and vegetation mortality. Plant Physiol 155:1051–1059. 21239620 10.1104/pp.110.170704 PMC3046567

[154] Medvigy D, Wofsy SC, Munger JW, Hollinger DY, Moorcroft PR (2009) Mechanistic scaling of ecosystem function and dynamics in space and time: ecosystem demography model version 2. J Geophys Res 114: 1–21. http://doi.wiley.com/10.1029/2008JG000812, (8 December 2018, date last accessed).

[156] Meier IC, Leuschner C (2008) Belowground drought response of European beech: fine root biomass and carbon partitioning in 14 mature stands across a precipitation gradient. Glob Chang Biol 14:2081–2095.

[158] Meyer A, Grote R, Polle A, Butterbach-Bahl K (2009) Simulating mycorrhiza contribution to forest C- and N cycling-the MYCOFON model. Plant Soil 327:493–517.

[157] Meyer A, Grote R, Butterbach-Bahl K (2012) Integrating mycorrhiza in a complex model system: effects on ecosystem C and N fluxes. Eur J For Res 131:1809–1831.

[159] Michelot A, Simard S, Rathgeber C, Dufrene E, Damesin C (2012) Comparing the intra-annual wood formation of three European species (*Fagus sylvatica*, *Quercus petraea* and *Pinus sylvestris*) as related to leaf phenology and non-structural carbohydrate dynamics. Tree Physiol 32:1033–1045. 22718524 10.1093/treephys/tps052

[160] Mildner M, Bader MK-F, Leuzinger S, Siegwolf RTW, Körner C (2014) Long-term 13C labeling provides evidence for temporal and spatial carbon allocation patterns in mature *Picea abies*. Oecologia 175:747–762. 24696359 10.1007/s00442-014-2935-5

[161] Monks A, Kelly D (2006) Testing the resource-matching hypothesis in the mast seeding tree *Nothofagus truncata* (Fagaceae). Austral Ecol 31:366–375.

[162] Monks A, Monks JM, Tanentzap AJ (2016) Resource limitation underlying multiple masting models makes mast seeding sensitive to future climate change. New Phytol 210:419–430. 26725252 10.1111/nph.13817

[163] Montané F, Fox AM, Arellano AF et al. (2017) Evaluating the effect of alternative carbon allocation schemes in a land surface model (CLM4.5) on carbon fluxes, pools, and turnover in temperate forests. Geosci Model Dev 10:3499–3517.

[164] Moore JAM, Jiang J, Post WM, Classen AT (2015) Decomposition by ectomycorrhizal fungi alters soil carbon storage in a simulation model. Ecosphere 6:art29.

[165] Muller B, Pantin F, Génard M, Turc O, Freixes S, Piques M, Gibon Y (2011) Water deficits uncouple growth from photosynthesis, increase C content, and modify the relationships between C and growth in sink organs. J Exp Bot 62:1715–1729. 21239376 10.1093/jxb/erq438

[166] Müller-Haubold H, Hertel D, Leuschner C (2015) Climatic drivers of mast fruiting in European beech and resulting C and N allocation shifts. Ecosystems 18:1083–1100.

[167] Murray MB, Smith RI, Friend A, Jarvis PG (2000) Effect of elevated [CO2] and varying nutrient application rates on physiology and biomass accumulation of Sitka spruce (*Picea sitchensis*). Tree Physiol 20:421–434. 12651438 10.1093/treephys/20.7.421

[168] Naudts K, Ryder J, McGrath MJ et al. (2015) A vertically discretised canopy description for ORCHIDEE (SVN r2290) and the modifications to the energy, water and carbon fluxes. Geosci Model Dev 8:2035–2065.

[169] Nielsen CN, Knudsen MA (2004) Stormstabilitet og sundhed i en rødgranskærm. D S T 89:115–128.

[170] Nikolova PS, Andersen CP, Blaschke H, Matyssek R, Häberle K-H (2010) Belowground effects of enhanced tropospheric ozone and drought in a beech/spruce forest (*Fagus sylvatica* L./*Picea abies* [L.] Karst). Environ Pollut 158:1071–1078. 19682778 10.1016/j.envpol.2009.07.036

[171] Oleson KW, Lawrence DM, Bonan GB et al. (2013) Technical description of version 4.5 of the Community Land Model (CLM), NCAR Technical Note No. NCAR/TN-503+STR. doi:10.5065/D6RR1W7M.

[172] Ostle NJ, Smith P, Fisher R et al. (2009) Integrating plant–soil interactions into global carbon cycle models. J Ecol 97:851–863.

[173] Ostrogović Sever MZ, Paladinić E, Barcza Z, Hidy D, Kern A, Anić M, Marjanović H (2017) Biogeochemical modelling vs. tree-ring measurements-comparison of growth dynamic estimates at two distinct oak forests in Croatia. South-east European Forestry. 8: 71–84.

[174] Overdieck D, Ziche D, Bottcher-Jungclaus K (2007) Temperature responses of growth and wood anatomy in European beech saplings grown in different carbon dioxide concentrations. Tree Physiol 27:261–268. 17241968 10.1093/treephys/27.2.261

[175] Palacio S, Hester AJ, Maestro M, Millard P (2008) Browsed *Betula pubescens* trees are not carbon-limited. Funct Ecol 22:808–815.

[176] Palacio S, Paterson E, Sim A, Hester AJ, Millard P (2011) Browsing affects intra-ring carbon allocation in species with contrasting wood anatomy. Tree Physiol 31:150–159. 21388994 10.1093/treephys/tpq110

[177] Pappas C, Fatichi S, Leuzinger S, Wolf A, Burlando P (2013) Sensitivity analysis of a process-based ecosystem model: pinpointing parameterization and structural issues. J Geophys Res Biogeo 118:505–528.

[178] Parton WJ, Schimel DS, Cole CV, Ojima DS (1987) Analysis of factors controlling soil organic matter levels in great plains grasslands 1. Soil Sci Soc Am J 51:1173–1179.

[179] Pavlick R, Drewry DT, Bohn K, Reu B, Kleidon A (2013) The Jena diversity-dynamic global vegetation model (JeDi-DGVM): a diverse approach to representing terrestrial biogeography and biogeochemistry based on plant functional trade-offs. Biogeosciences 10:4137–4177.

[180] Pearse IS, Koenig WD, Kelly D (2016) Mechanisms of mast seeding: resources, weather, cues, and selection. New Phytol 212:546–562. 27477130 10.1111/nph.14114

[181] Perttunen J, Sievänen R, Nikinmaa E (1998) LIGNUM: a model combining the structure and the functioning of trees. Ecol Model 108:189–198.

[182] Peters EB, Wythers KR, Zhang S, Bradford JB, Reich PB (2013) Potential climate change impacts on temperate forest ecosystem processes. Can J For Res 43:939–950.

[183] Pezzatti G (2011) Modeling plant biomass partitioning: responses to environmental conditions and disturbance. http://www.fedoa.unina.it/8515/. (8 December 2018, date last accessed).

[184] Pianosi F, Beven K, Freer J, Hall JW, Rougier J, Stephenson DB, Wagener T (2016) Sensitivity analysis of environmental models: a systematic review with practical workflow. Environ Model Softw 79:214–232.

[185] Pinkard EA, Battaglia M, Roxburgh S, O’Grady AP (2011) Estimating forest net primary production under changing climate: adding pests into the equation. Tree Physiol 31:686–699. 21746746 10.1093/treephys/tpr054

[186] Piovesan G, Adams JM (2001) Masting behaviour in beech: linking reproduction and climatic variation. Can J Bot 79:1039–1047.

[187] Piovesan G, Adams JM (2005) The evolutionary ecology of masting: does the environmental prediction hypothesis also have a role in Mesic temperate forests? Ecol Res 20:739–743.

[188] Piper FI, Gundale MJ, Fajardo A (2015) Extreme defoliation reduces tree growth but not C and N storage in a winter-deciduous species. Ann Bot 115:1093–1103. 25851136 10.1093/aob/mcv038 PMC4648455

[189] Poorter H, Niklas KJ, Reich PB, Oleksyn J, Poot P, Mommer L (2011) Biomass allocation to leaves, stems and roots: meta-analyses of interspecific variation and environmental control. New Phytol 193:30–50. 22085245 10.1111/j.1469-8137.2011.03952.x

[190] Pretzsch H (2009) Forest dynamics, growth, and yield, Berlin Heidelberg: Springer-Verlag, pp. 1–39.

[191] Pretzsch H, Forrester DI, Rötzer T (2015) Representation of species mixing in forest growth models. A review and perspective. Ecol Model 313:276–292.

[192] Pumpanen J, Heinonsalo J, Rasilo T, Villemot J, Ilvesniemi H (2012) The effects of soil and air temperature on CO2 exchange and net biomass accumulation in Norway spruce, Scots pine and silver birch seedlings. Tree Physiol 32:724–736. 22345325 10.1093/treephys/tps007

[193] Quentin AG, Pinkard EA, Ryan MG et al. (2015) Non-structural carbohydrates in woody plants compared among laboratories. Tree Physiol 35:1146–1165. 26423132 10.1093/treephys/tpv073

[194] Repola J (2008) Biomass equations for birch in Finland. Silva Fenn 42: 605–624. 10.14214/sf.236. (8 December 2018, date last accessed).

[195] Reyer CPO, Bathgate S, Blennow K et al. (2017) Are forest disturbances amplifying or canceling out climate change-induced productivity changes in European forests? Environ Res Lett 12:034027. 28855959 10.1088/1748-9326/aa5ef1 PMC5572643

[196] Richardson AD, Carbone MS, Huggett BA, Furze ME, Czimczik CI, Walker JC, Xu X, Schaberg PG, Murakami P (2015) Distribution and mixing of old and new nonstructural carbon in two temperate trees. New Phytol 206:590–597. 25558814 10.1111/nph.13273 PMC4405048

[197] Robinson AP, Ek AR (2000) The consequences of hierarchy for modeling in forest ecosystems. Can J For Res 30:1837–1846.

[198] Rolo V, Andivia E, Pokorný R (2015) Response of *Fagus sylvatica* and *Picea abies* to the interactive effect of neighbor identity and enhanced CO2 levels. Trees 29:1459–1469.

[199] Rötzer T, Leuchner M, Nunn AJ (2010) Simulating stand climate, phenology, and photosynthesis of a forest stand with a process-based growth model. Int J Biometeorol 54:449–464. 20084520 10.1007/s00484-009-0298-0

[200] Rötzer T, Seifert T, Gayler S, Priesack E, Pretzsch H (2012) Effects of stress and defence allocation on tree growth: simulation results at the individual and stand level In: Matyssek R, Schnyder H, Oßwald W, Ernst D, Munch JC, Pretzsch H (eds) Growth and defence in plants: resource allocation at multiple scales. Springer, Berlin, Heidelberg, pp 401–432. 10.1007/978-3-642-30645-7_18.

[201] Running S, Hunt E (1993) Generalization of a Forest ecosystem process model for other biomes, BIOME-BCG, and an application for global-scale models In: Ehleringer JR, Field CB (eds.) Scaling physiological processes: leaf to globe: a volume in physiological ecology, San Diego: Academic Press, Inc. pp 141–158.

[202] Running SW (2008) Climate change. Ecosystem disturbance, carbon, and climate. Science 321:652–653. 18669853 10.1126/science.1159607

[203] Running SW, Gower ST (1991) FOREST-BGC, a general model of forest ecosystem processes for regional applications. II. Dynamic carbon allocation and nitrogen budgets. Tree Physiol 9:147–160. 14972861 10.1093/treephys/9.1-2.147

[204] Ruotsalainen A, Tuomi J, Väre H (2002) A model for optimal mycorrhizal colonization along altitudinal gradients. Silva Fenn 36: 681–694. 10.14214/sf.533. (8 December 2018, date last accessed).

[205] Ryan MG, Stape JL, Binkley D et al. (2010) Factors controlling eucalyptus productivity: how water availability and stand structure alter production and carbon allocation. For Ecol Manage 259:1695–1703.

[206] Saffell BJ, Meinzer FC, Woodruff DR, Shaw DC, Voelker SL, Lachenbruch B, Falk K (2014) Seasonal carbohydrate dynamics and growth in Douglas-fir trees experiencing chronic, fungal-mediated reduction in functional leaf area. Tree Physiol 34:218–228. 24550088 10.1093/treephys/tpu002

[207] Sala A, Hoch G (2009) Height-related growth declines in ponderosa pine are not due to carbon limitation. Plant Cell Environ 32:22–30. 19021883 10.1111/j.1365-3040.2008.01896.x

[209] Sala A, Woodruff DR, Meinzer FC (2012 *a*) Carbon dynamics in trees: feast or famine? Tree Physiol 32:764–775. 22302370 10.1093/treephys/tpr143

[208] Sala A, Hopping K, McIntire EJB, Delzon S, Crone EE (2012 *b*) Masting in whitebark pine (*Pinus albicaulis*) depletes stored nutrients. New Phytol 196:189–199. 22889129 10.1111/j.1469-8137.2012.04257.x

[210] Sánchez-Salguero R, Camarero JJ, Gutiérrez E, González Rouco F, Gazol A, Sangüesa-Barreda G, Andreu-Hayles L, Linares JC, Seftigen K (2016) Assessing forest vulnerability to climate warming using a process-based model of tree growth: bad prospects for rear-edges. Glob Chang Biol 23:2705–2719. 27782362 10.1111/gcb.13541

[211] Satake A, Iwasa Y (2000) Pollen coupling of forest trees: forming synchronized and periodic reproduction out of chaos. J Theor Biol 203:63–84. 10704293 10.1006/jtbi.1999.1066

[212] Schaefer K, Collatz GJ, Tans P, Denning AS, Baker I, Berry J, Prihodko L, Suits N, Philpott A (2008) Combined simple biosphere/Carnegie-Ames-Stanford approach terrestrial carbon cycle model. J Geophys Res 113: 1–13. 10.1029/2007jg000603. (6 December 2018, date last accessed).

[213] Scheffer M, Carpenter S, Foley JA, Folke C, Walker B (2001) Catastrophic shifts in ecosystems. Nature 413:591–596. 11595939 10.1038/35098000

[214] Scheller R, Hua D, Bolstad P, Birdsey RA, Mladenoff D (2011) The effects of forest harvest intensity in combination with wind disturbance on carbon dynamics in lake states mesic forests. Ecological Modelling. 222: 144–153 DOI: 10.1016/j.ecolmodel.2010.09.009.

[215] Schiestl-Aalto P, Kulmala L, Mäkinen H, Nikinmaa E, Mäkelä A (2015) CASSIA - a dynamic model for predicting intra-annual sink demand and interannual growth variation in Scots pine. New Phytol 206:647–659. 25616175 10.1111/nph.13275

[216] Schiestl-Aalto P, Mäkelä A (2017) Temperature dependence of needle and shoot elongation before bud break in scots pine. Tree Physiol. 37: 316–325. 10.1093/treephys/tpw120. (8 December 2018, date last accessed). 28008084

[217] Schiestl-Aalto P, Ryhti K, Mäkelä A, Peltoniemi M, Bäck J, Kulmala L (2019) Analysis of the NSC storage dynamics in tree organs reveals the allocation to belowground Symbionts in the framework of whole tree carbon balance. Front For Glob Change 2: 1–17. https://www.frontiersin.org/articles/10.3389/ffgc.2019.00017/full. (2 June 2019, date last accessed).

[218] Schippers P, Vlam M, Zuidema PA, Sterck F (2015) Sapwood allocation in tropical trees: a test of hypotheses. Funct Plant Biol 42:697. 10.1071/FP14127 32480713

[220] Seidl R, Blennow K (2012) Pervasive growth reduction in Norway spruce forests following wind disturbance. PLoS One 7:e33301. 22413012 10.1371/journal.pone.0033301 PMC3296682

[222] Seidl R, Lexer MJ, Jäger D, Hönninger K (2005) Evaluating the accuracy and generality of a hybrid patch model. Tree Physiol 25:939–951. 15870060 10.1093/treephys/25.7.939

[219] Seidl R, Baier P, Rammer W, Schopf A, Lexer MJ (2007) Modelling tree mortality by bark beetle infestation in Norway spruce forests. Ecol Model 206:383–399.

[223] Seidl R, Rammer W, Bellos P, Hochbichler E, Lexer MJ (2009) Testing generalized allometries in allocation modeling within an individual-based simulation framework. Trees 24:139–150.

[221] Seidl R, Fernandes PM, Fonseca TF et al. (2011) Modelling natural disturbances in forest ecosystems: a review. Ecol Model 222:903–924.

[224] Seidl R, Rammer W, Scheller RM, Spies TA (2012) An individual-based process model to simulate landscape-scale forest ecosystem dynamics. Ecol Model 231:87–100.

[225] Seidl R, Schelhaas M-J, Rammer W, Verkerk PJ (2014) Increasing forest disturbances in Europe and their impact on carbon storage. Nat Clim Chang 4:806–810. 25737744 10.1038/nclimate2318 PMC4340567

[226] Seidl R, Thom D, Kautz M et al. (2017) Forest disturbances under climate change. Nat Clim Chang 7:395–402. 28861124 10.1038/nclimate3303 PMC5572641

[227] Selås V, Piovesan G, Adams JM, Bernabei M (2002) Climatic factors controlling reproduction and growth of Norway spruce in southern Norway. Can J For Res 32:217–225.

[228] Sevanto S, Dickman LT (2015) Where does the carbon go? Plant carbon allocation under climate change. Tree Physiol 35:581–584. 26109074 10.1093/treephys/tpv059

[229] Shinozaki K, Yoda K, Hozumi K, Kira T (1964) A quantitative analysis of planr form – the pipe model theory: I. Basic Analyses 14:97–105.

[230] Sievänen R, Perttunen J, Nikinmaa E, Kaitaniemi P (2008) Toward extension of a single tree functional–structural model of scots pine to stand level: effect of the canopy of randomly distributed, identical trees on development of tree structure. Funct Plant Biol 35:964–975. 10.1071/FP08077 32688846

[231] Sitch S, Smith B, Prentice IC et al. (2003) Evaluation of ecosystem dynamics, plant geography and terrestrial carbon cycling in the LPJ dynamic global vegetation model. Glob Chang Biol 9:161–185.

[232] Smith B, Prentice IC, Sykes MT (2001) Representation of vegetation dynamics in the modelling of terrestrial ecosystems: comparing two contrasting approaches within European climate space. Glob Ecol Biogeogr 10:621–637.

[233] Smith B, Wårlind D, Arneth A, Hickler T, Leadley P, Siltberg J, Zaehle S (2014) Implications of incorporating N cycling and N limitations on primary production in an individual-based dynamic vegetation model. Biogeosciences 11:2027–2054.

[234] Svensson M, Jansson P-E, Kleja DB (2008) Modelling soil C sequestration in spruce forest ecosystems along a Swedish transect based on current conditions. Biogeochemistry 89:95–119.

[235] Thomas CK, Law BE, Irvine J, Martin JG, Pettijohn JC, Davis KJ (2009) Seasonal hydrology explains interannual and seasonal variation in carbon and water exchange in a semiarid mature ponderosa pine forest in Central Oregon. J Geophys Res 114: 1–22. 10.1029/2009jg001010. (8 December 2018, date last accessed).

[236] Thornton P, Running SW, Hunt ER (2005) Biome-BGC: terrestrial ecosystem process model, version 4.1.1.

[237] Ulrich B (1993) Prozeßhierarchie in Waldökosystemen. Ein integrierender okosystemtheoretischer Ansatz. Biologie in unserer Zeit 23:322–329.

[238] Usami T, Lee J, Oikawa T (2001) Interactive effects of increased temperature and CO2 on the growth of *Quercus myrsinaefolia* saplings. Plant Cell Environ 24:1007–1019.

[240] Vacchiano G, Hacket-Pain A, Turco M, Motta R, Maringer J, Conedera M, Drobyshev I, Ascoli D (2017) Spatial patterns and broad-scale weather cues of beech mast seeding in Europe. New Phytol 215:595–608. 28631320 10.1111/nph.14600

[239] Vacchiano G, Ascoli D, Berzaghi F et al. (2018) Reproducing reproduction: how to simulate mast seeding in forest models. Ecol Model 376:40–53.

[241] Valentine HT, Mäkelä A (2005) Bridging process-based and empirical approaches to modeling tree growth. Tree Physiol 25:769–779. 10.1093/treephys/25.7.769 15870047

[242] Valentine HT, Mäkelä A (2012) Modeling forest stand dynamics from optimal balances of carbon and nitrogen. New Phytol 194:961–971. 22463713 10.1111/j.1469-8137.2012.04123.x

[155] van der Meer PJ, Jorritsma ITM, Kramer K (2002) Assessing climate change effects on long-term forest development: adjusting growth, phenology, and seed production in a gap model. For Ecol Manage 162:39–52.

[243] Van Oijen M, Rougier J, Smith R (2005) Bayesian calibration of process-based forest models: bridging the gap between models and data. Tree Physiol 25:915–927. 15870058 10.1093/treephys/25.7.915

[244] Vanninen P, Mäkelä A (2005) Carbon budget for scots pine trees: effects of size, competition and site fertility on growth allocation and production. Tree Physiol 25:17–30. 15519982 10.1093/treephys/25.1.17

[245] Vargas R (2009) On the fate of old stored carbon after large-infrequent disturbances in plants. Plant Signal Behav 4:617–619. 19820352 10.4161/psb.4.7.8906 PMC2710554

[246] Vargas R, Trumbore SE, Allen MF (2009) Evidence of old carbon used to grow new fine roots in a tropical forest. New Phytol 182:710–718. 19434807 10.1111/j.1469-8137.2009.02789.x

[247] Venner S, Siberchicot A, Pélisson P-F et al. (2016) Fruiting strategies of perennial plants: a resource budget model to couple mast seeding to pollination efficiency and resource allocation strategies. Am Nat 188:66–75. 27322122 10.1086/686684

[248] Vermeulen MH, Kruijt BJ, Hickler T, Kabat P (2015) Modelling short-term variability in carbon and water exchange in a temperate Scots pine forest. Earth Syst Dynam 6:485–503.

[249] Vicca S, Luyssaert S, Peñuelas J et al. (2012) Fertile forests produce biomass more efficiently. Ecol Lett 15:520–526. 22472207 10.1111/j.1461-0248.2012.01775.x

[250] Vilà-Cabrera A, Martínez-Vilalta J, Retana J (2014) Variation in reproduction and growth in declining scots pine populations. Pers Plant Ecol Evol Syst 16:111–120.

[251] Wardlaw IF (1990) Tansley review no. 27 the control of carbon partitioning in plants. New Phytol 116:341–381. 10.1111/j.1469-8137.1990.tb00524.x 33874094

[252] Wårlind D, Smith B, Hickler T, Arneth A (2014) Nitrogen feedbacks increase future terrestrial ecosystem carbon uptake in an individual-based dynamic vegetation model. Biogeosciences 11:6131–6146.

[253] Warnant P, FrançOis L, Strivay D, GéRard J-C (1994) CARAIB: a global model of terrestrial biological productivity. Global Biogeochem Cycles 8:255–270.

[254] Warren JM, Iversen CM, Garten CT et al. (2011) Timing and magnitude of C partitioning through a young loblolly pine (*Pinus taeda* L.) stand using 13C labeling and shade treatments. Tree Physiol 32:799–813. 22210530 10.1093/treephys/tpr129

[255] Way DA, Oren R (2010) Differential responses to changes in growth temperature between trees from different functional groups and biomes: a review and synthesis of data. Tree Physiol 30:669–688. 20368338 10.1093/treephys/tpq015

[256] White MA, Thornton PE, Running SW (1997) A continental phenology model for monitoring vegetation responses to interannual climatic variability. Global Biogeochem Cycles 11:217–234.

[257] White MA, Thornton PE, Running SW, Nemani RR (2000) Parameterization and sensitivity analysis of the BIOME–BGC terrestrial ecosystem model: net primary production controls. Earth Interact 4:1–85.

[258] Wieser G, Matyssek R (2007) Linking ozone uptake and defense towards a mechanistic risk assessment for forest trees. New Phytol 174:7–9. 17335492 10.1111/j.1469-8137.2007.01994.x

[259] Wiley E, Huepenbecker S, Casper BB, Helliker BR (2013) The effects of defoliation on carbon allocation: can carbon limitation reduce growth in favour of storage? Tree Physiol 33:1216–1228. 24271085 10.1093/treephys/tpt093

[261] Wolf A, Ciais P, Bellassen V, Delbart N, Field CB, Berry JA (2011) Forest biomass allometry in global land surface models. Global Biogeochem Cycles 25: 1–16.

[262] Xia J, Yuan W, Wang Y-P, Zhang Q (2017) Adaptive carbon allocation by plants enhances the terrestrial carbon sink. Sci Rep 7: 1–11. 10.1038/s41598-017-03574-3. (8 December 2018, date last accessed). 28611453 PMC5469799

[263] Yan Z, Li P, Chen Y, Han W, Fang J (2016) Nutrient allocation strategies of woody plants: an approach from the scaling of nitrogen and phosphorus between twig stems and leaves. Sci Rep 6: 1–9, 10.1038/srep20099, (8 December 2018, date last accessed). 26848020 PMC4742826

[264] Zaehle S (2013) Terrestrial nitrogen-carbon cycle interactions at the global scale. Philos Trans R Soc Lond B Biol Sci 368:20130125. 23713123 10.1098/rstb.2013.0125 PMC3682745

