# Supplementary material for: Forest carbon allocation modelling under climate change
Source: Tree Physiol. 2019 Nov 21;39(12):1937–60. doi: 10.1093/treephys/tpz105 (PMC6995853; doi:10.1093/treephys/tpz105)
Supplement: Merganicova_etal_CarbonAllocation_17092019_Suplement_tpz105 [file merganicova_etal_carbonallocation_17092019_suplement_tpz105.docx]

# Supplementary

## Questionnaire

### 1. General information about the whole modelling system

1.1. Name of the model * (the whole modelling system)

1.1.1. Name of the subsystem / module, which comprises a carbon allocation model you will describe below

If the whole modelling system consists of several different modules used in specific cases, e.g. in different vegetation types, please indicate the name of the subsystem, in which the described carbon allocation model is incorporated.

1.1.2. Ecosystems that can be simulated by the subsystem / module you entered in question 1.1.1. (or the whole modelling system if you did not answer question 1.1.1.) *

- forest
- arable land
- grassland
- shrubland
- wetland
- C3 plants
- C4 plants
- Other:

1.2. Modelling concept of the subsystem / module, which comprises the carbon allocation model you are going to describe below *

Please, tick one or more appropriate options

- hybrid (combination of any below-listed concepts) - please tick the concepts that are used in the model
- empirical (based on statistical relationships derived from empirical data)
- structural (development of tree morphology)
- physiological / process-based (based on mathematical description of processes in ecosystems)
- theoretical (data-free concept based on mathematical or physical theories)

1.2.1. Predominant modelling concept

If your system/subsystem uses a hybrid approach, tick the basic approach the system is built upon

- empirical
- structural
- physiological / process-based
- theoretical

1.3. Modelled object *

The minimum representative level of your modelling system. (= the object that the applied mathematical algorithms and input state variables are related to.)

- ecosystem (e.g.biome)
- population (e.g. forest stand)
- class / cohort (e.g. diameter class)
- organism / individual / tree
- organ (e.g. leaf)

1.4. Modelled spatial scale *

- global
- landscape
- region
- forest stand
- biogroup (a group of trees in a specific developmental phase covering an area of 100 to 1,000 m2. A biogroup can consist of several cohorts or size classes.)
- cohort / size class (a group of trees with identical properties, e.g. size, species)
- modelled object located in 2D space (e.g. tree or organ)
- modelled object located in 3D space
- Other:

1.5. Prevailing spatial unit simulated by the modelling system/subsystem

If you specified more than one spatial scale in the previous question, please indicate the scale for which the model is used most frequently

- global
- landscape
- region
- forest stand
- biogroup
- cohort / size class
- modelled object located in 2D space
- modelled object located in 3D space

1.6. Resolution of minimum spatial unit

Please indicate an area or pixel size (e.g. 100x100m)

1.7. Shortest model time step *

that your modelling system is able to simulate

- millenium
- century
- decade
- 5 years
- year
- month
- day
- hour
- minute
- Other:

1.8. Applicable region *

Please tick all regions for which the model was parameterised

- Boreal
- Temperate
- Mediterranean
- Tropical

1.9. The country of model origin *

1.10. Number of carbon allocation models incorporated in one subsystem / module *

Please specify how many carbon allocation models are incorporated in the subsystem, e.g. if the subsystem uses different carbon allocation models for C3 and C4 plants, the answer is 2

### 2. Information about carbon allocation modelling

If your modelling system comprises more carbon allocation models used in specific conditions, please fill in this part for each carbon allocation model separately.

2.1. Principles of carbon allocation modelling *

(based on de Kauwe et al. 2014 and Franklin et al. 2012)

- empirical approach (based on statistically described relationships)
- functional relationship (based on the scaling relationships among plant organs)
- functional-balance approach (allocation is controlled to ensure internal balance among organs, e.g. root vs. shoot growth)
- eco-evolutionarily-based approach (allocation is determined by maximising a fitness proxy, e.g. photosynthesis, NPP)
- thermodynamic approach (maximisation of entropy / entropy production)
- Other:

2.2. Type of carbon allocation modelling *

(based on de Kauwe et al. 2014, Franklin et al. 2012, Fabrika and Pretzsch 2011, Lacointe 2000)

- fixed ratios (fixed fractions of assimilated carbon are allocated to individual organs)
- allometry (growth of an organ is related to the growth of the whole organism or its other part)
- teleonomic (functional) balance of root/shoot activities
- resource limitation (allocation depends on the most limiting resource to growth)
- pipe model (based on the balance between foliage and sapwood)
- mechanical constraints (allocation of biomass along the stem ensures mechanical stability of a tree)
- transport-resistance model (allocation is driven by concentration of elements: carbon, nitrogen)
- source-sink model (allocation to individual compartments is controlled by their demands and the availability of assimilates)
- optimal response (maximisation of a fitness proxy, e.g. photosynthesis, with respect to functional traits, e.g. stomatal conductance, subject to environmental and/or physiological constraints, e.g. N balance)
- game-theoretic optimisation (based on the concept of an evolutionary stable strategy, when the success of each individual depends on the competition with other individuals)
- adaptive dynamics (based on the concept of an evolutionary stable strategy, the allocation at the individual level evolves through the effect of selection via explicit modeling of population dynamics)
- maximum entropy production (based on thermodynamics, which identifies the most likely allocation considering the state of population)
- Other:

2.3. Time step of the carbon allocation model *

- minute
- hour
- day
- month
- year
- 5 years
- decade
- century
- millennium
- Other:

2.4. Spatial scale of the carbon allocation model *

Please specify, at what spatial level allocation occurs.

- tree
- cohort
- stand
- region
- biome
- Other:

2.5. Parameters affecting carbon available for allocation *

Please indicate the parameters that modify GPP prior to the allocation itself. Do not indicate the variables driving GPP, but specify what parameters affect the total amount of carbon that is available for the allocation. Use semicolon (;) to separate multiple entries under "Other" option.

- growth respiration
- maintenance respiration
- temperature
- CO2 concentration
- light availability
- nitrogen availability
- water availability
- availability of other nutrients - please specify which nutrients in question 2.5.1.
- disturbance - please specify the type of disturbances in question 2.5.2.
- phenology
- no
- Other:

2.5.1 What nutrients affect carbon available for allocation?

Please indicate the nutrients that modify GPP prior to the allocation itself. Use semicolon (;) to separate multiple entries under "Other" option.

- P (Phosphorus)
- K (Potassium)
- Mg (Magnesium)
- Other:

2.5.2. What disturbances affect carbon available for allocation?

Please indicate the disturbances that modify GPP prior to the allocation itself. Use semicolon (;) to separate multiple entries under "Other" option.

- wind
- fire
- insects
- drought
- Other:

2.6. Individual compartments for carbon allocation *

Please specify the smallest pools your model uses in the allocation algorithm. If your model identifies more detailed or lumped compartments not specified below, e.g. stem+branches+roots, please indicate that under "Other" option. Use semicolon (;) to separate multiple entries under "Other" option.

- leaf
- live stem / sap wood
- dead stem / heart wood
- stem (sap wood + heart wood)
- live coarse roots
- dead coarse root
- coarse root (live + dead coarse root)
- fine root
- root (coarse root + fine root)
- branch
- crown (branch + twig + leaf)
- flower
- pollen
- fruit (including seeds)
- seed
- storage / reserve
- vegetative reproduction
- stem + crown
- stem + branch
- branch + root
- branch + coarse root
- aboveground carbon (leaf + stem + crown)
- belowground carbon (coarse root + fine root)
- tree diameter
- tree height
- tree volume
- diameter increment
- height increment
- volume increment
- Other:

2.7. Constant parameters *

Please specify, which carbon allocation parameters / coefficients are kept constant during a single simulation. Use semicolon (;) to separate multiple entries under "Other" option.

- C:N ratios of individual compartments
- compartment fractions (i.e. parameters specifying the proportion of carbon allocated to each compartment)
- compartment allocation ratios (i.e. ratio of allocated carbon between two compartments, e.g. ratio between carbon allocated to new stem and carbon allocated to new leaf)
- allometric coefficients (i.e. coefficients of allometric relationships)
- fraction of growth respiration
- fraction of maintenance respiration
- growth proportion
- reproduction fraction
- Other:

2.7.1. Can the constant parameters be changed by a model user for different simulations? *

- No, they are defined in the source code
- Yes, they can be changed e.g. in input box, file
- Some parameters can be changed externally - please specify below in question 2.7.2.

2.7.2. Which constant parameters can be changed by a model user for different simulations?

Please answer this question if you selected the last answer on the previous question 2.7.1. Use semicolon (;) to separate multiple entries under "Other" option.

- C:N ratios of individual compartments
- compartment fractions
- compartment allocation ratios
- allometric coefficients
- fraction of growth respiration
- fraction of maintenance respiration
- growth proportion
- reproduction fraction
- Other:

2.8. Priority of carbon allocation to any compartments *

Please specify if carbon is allocated according to any pre-defined priorities.

- No
- Yes, please specify below in questions 2.8.1 to 2.8.3.

2.8.1. If carbon allocation is prioritised to any compartments, please specify the compartment of the 1st priority below

Use semicolon (;) to separate multiple entries under "Other" option.

- leaf
- stem
- root
- fruit
- fine root
- coarse root
- live stem / sapwood
- dead stem / heartwood
- Other:

2.8.2. If carbon allocation is prioritised to any compartments, please specify the compartment of the 2nd priority below

Use semicolon (;) to separate multiple entries under "Other" option.

- leaf
- stem
- root
- fruit
- fine root
- coarse root
- live stem / sapwood
- dead stem / heartwood
- Other:

2.8.3. If carbon allocation is prioritised to any compartments, please specify the compartment of the 3rd priority below

Use semicolon (;) to separate multiple entries under "Other" option.

- leaf
- stem
- root
- fruit
- fine root
- coarse root
- live stem / sap wood
- dead stem / heart wood
- Other:

2.8.4. If carbon allocation is prioritised to compartments depending on the phenological phase, please specify the phase and the compartment(s), which is prioritised in the specific phase

Example: leaf unfolding - leaf; leaf colouring - root. Use semicolon (;) to separate multiple entries.

2.8.5. If carbon allocation is prioritised to specific compartments depending on any other parameter, please specify the parameter and individual states of the parameter (if applicable) and the compartment(s), which is/are prioritised

Example: leaf damage by insects - leaf. Use semicolon (;) to separate multiple entries.

2.9. Sensitivity of the carbon allocation algorithm to *

Please tick what parameters drive carbon allocation (e.g. type of allocation, its parameters, coefficients, equations). This question does not refer to the sensitivity of GPP algorithm. Use semicolon (;) to separate multiple entries under "Other" option.

- air temperature
- precipitation
- CO2 concentration
- light availability
- soil water
- nitrogen
- soil nutrients - please specify them in question 2.9.1.
- competition
- leaf phenology
- size of the modelled object (e.g. tree)
- age of the modelled object (e.g. tree)
- compartment senescence (e.g. fine root mortality)
- tree species (group) / functional type - please specify them in question 2.9.2.
- genetics
- no
- Other:

2.9.1. What soil nutrients is the carbon allocation algorithm sensitive to?

Use semicolon (;) to separate multiple entries under "Other" option.

- P (Phosphorus)
- K (Potassium)
- Mg (Magnesium)
- Other:

2.9.2. If the carbon allocation model is sensitive to tree species / functional types, please specify the particular tree species (groups) or functional types the allocation model is applicable to

2.10. Was the carbon allocation model evaluated on data? *

- No
- Yes - please specify the data set below in question 2.11.

2.11. If the carbon allocation model was evaluated on data, please specify the data set and provide the reference (e.g. ICP Level II plots of Slovakia, Author, Year, Literature source)

2.12. Why was this carbon allocation model chosen? *

Please tick one or more appropriate reasons. Use semicolon (;) to separate multiple entries under "Other" option.

- Literature survey
- Expert opinion
- Model simplicity
- Data availability / requirements
- After the test of multiple carbon allocation models
- Sensitivity to environmental conditions
- I do not know
- Other:

2.13. Have you identified any problems of the implemented carbon allocation model? *

- No
- Yes - please specify them in question 2.13.1.

2.13.1. What problems of the implemented carbon allocation model have you identified?

### 3. General information

3.1. Reference - forest growth model (modelling system or subsystem) *

3.2. Has the implemented carbon allocation model been published? *

- No - please specify the reason in question 3.2.1.
- Yes - please provide the reference in question 3.2.2.

3.2.1. Why has the carbon allocation model not been published?

- It has not been validated
- It is a modification of another model - please specify it in question 3.2.2.
- Other:

3.2.2. Reference - carbon allocation model *

3.3. Reference - carbon allocation parameters *

3.4. May we contact you for further information if needed? *

- yes - please fill in questions 3.5. and 3.6.
- no

3.5. Your name

3.6. Your e-mail address

3.7. Please indicate whether you are *

- a model developer
- a model user

3.8. The country in which you are professionally active *

3.9. Comments

## Partial results


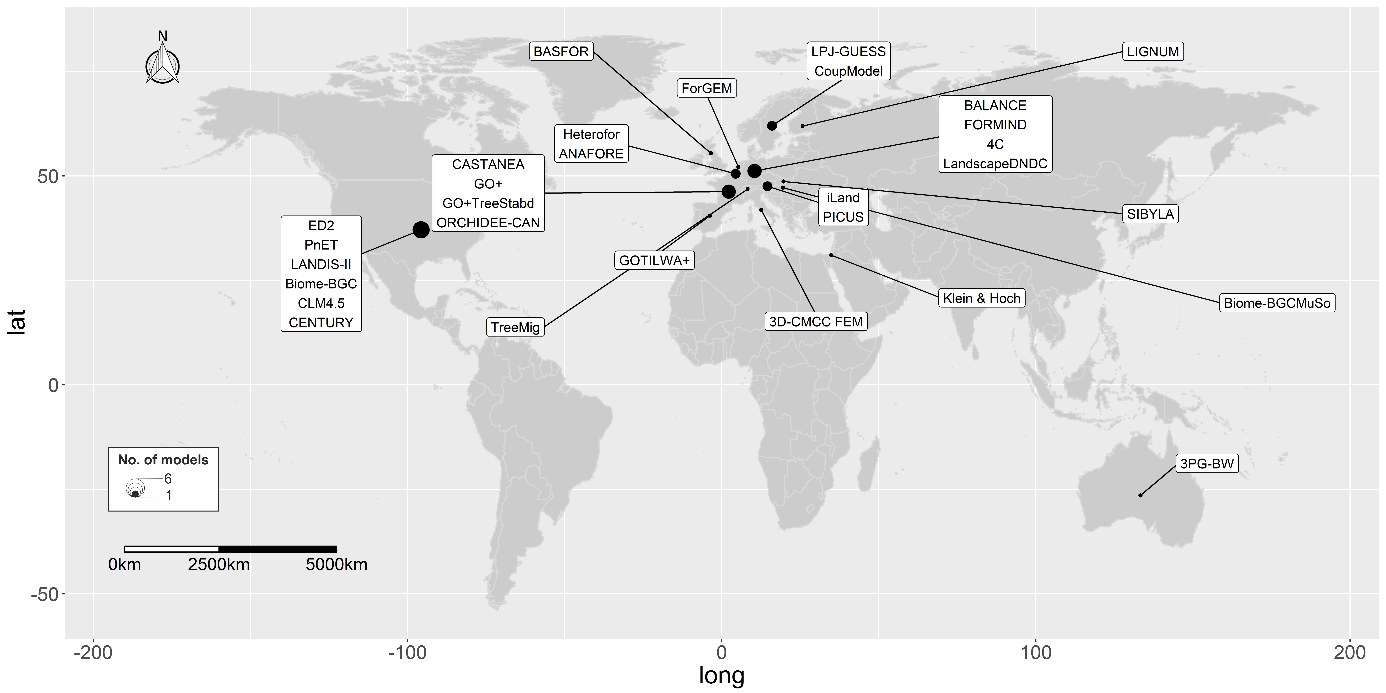


Figure S1. Distribution of the models included in our analysis on the base of their country of origin.


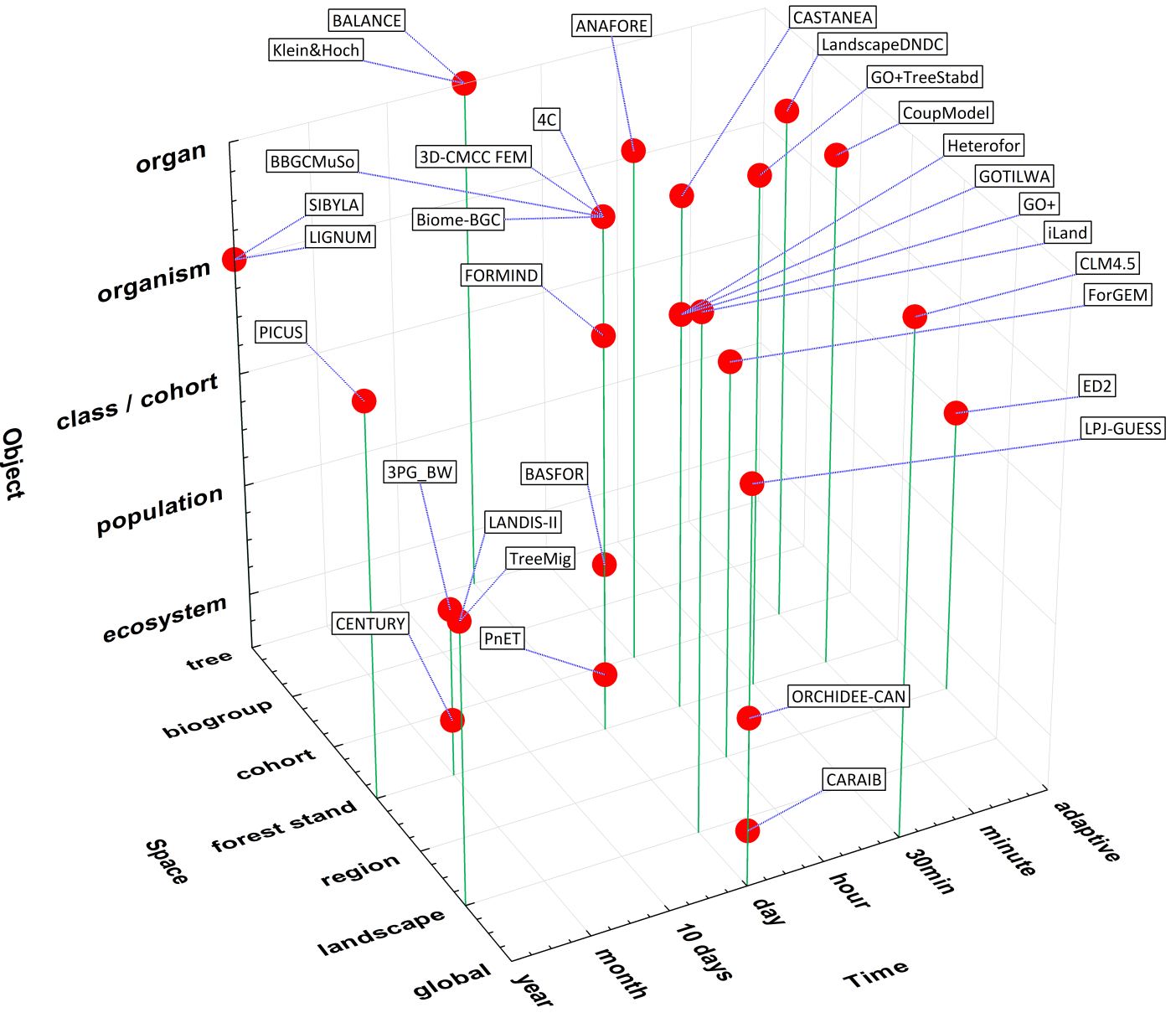


Figure S2. Model classification based on their temporal and spatial modelling scale and the modelled object.

A descriptive sensitivity analysis of carbon allocation models identified 17 properties which influence simulated carbon allocation in the examined models. We divided the factors into three main groups representing climatic conditions (4 factors), soil characteristics (4 factors), and plant or stand properties (9 factors). Although all models included at least one factor, none of them accounted for the direct impact of all the identified factors on modelled carbon allocation (Figure S1A). Only the factors from the group of plant characteristics were considered in every model from our database (Figure S1B). Only three models accounted for more than 50% of the factors (i.e. more than 8), while the majority of models (58%) included five or fewer factors and three models included only one factor (Figure S1A).

From plant characteristics, tree species or similar differentiation of vegetation (e.g. biomes, plant functional types, tree species groups) was the most common factor included in 23 models (74%, Figure 5). More than 50% of the models (16 models) accounted for the impact of leaf phenology or the size of the modelled object on the simulated carbon allocation, while wood phenology, genetics and the size of the allocated pool were considered only in one model each (Figure 5).


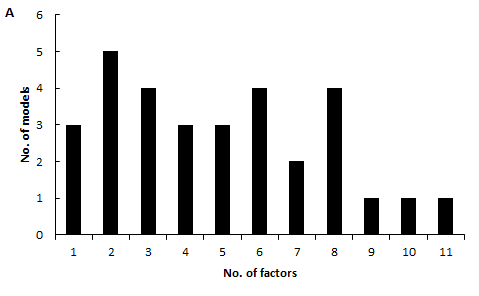


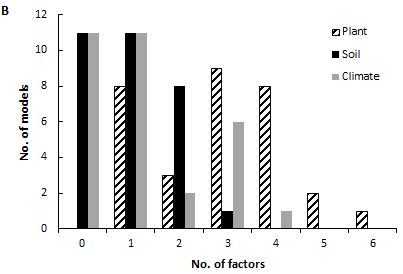


Figure S3. Frequency distribution of models with regard to number of environmental and stand/tree factors directly affecting simulated carbon allocation (A) divided into three main groups representing plant and stand characteristics, soil and climate conditions (B).


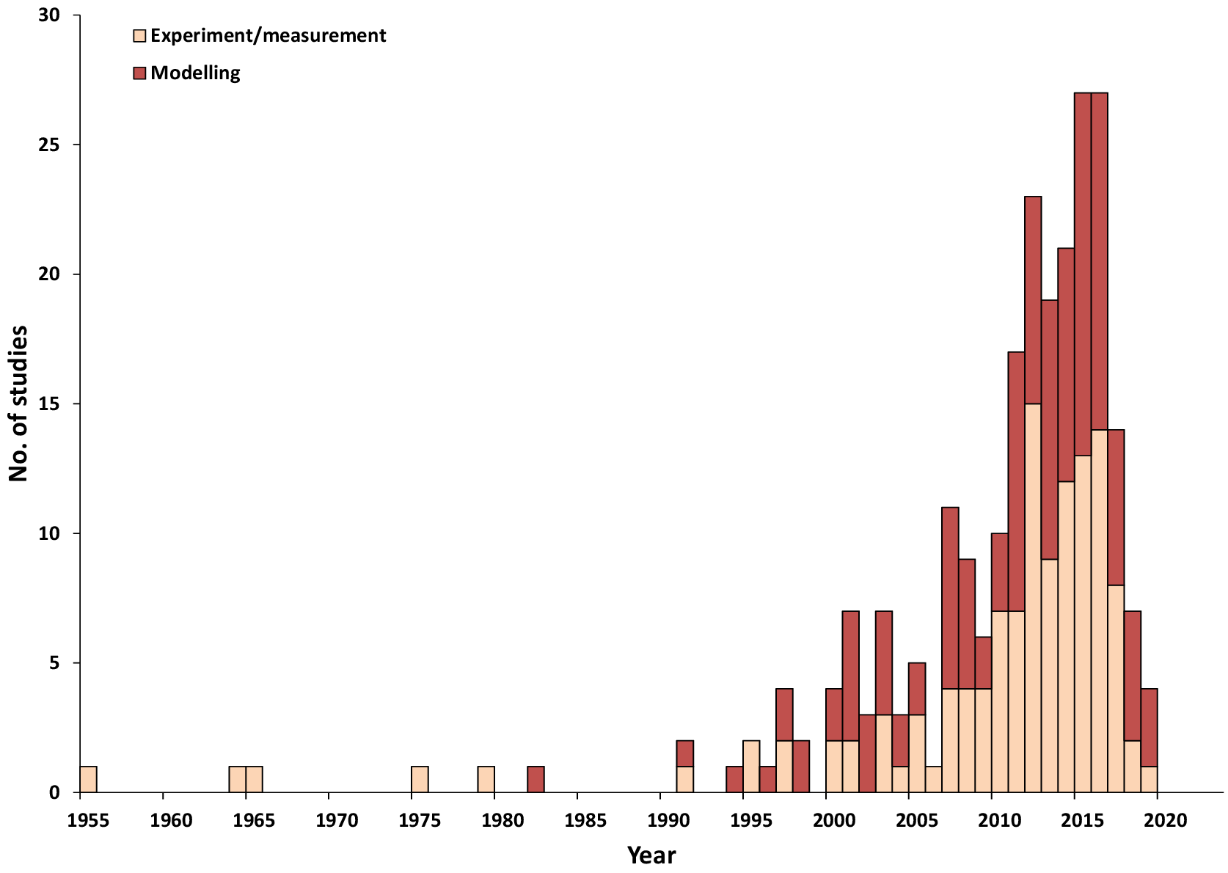


Figure S4. Temporal distribution of reviewed literature sources.
